# Two-layer detection framework with a high accuracy and efficiency for a malware family over the TLS protocol

**DOI:** 10.1371/journal.pone.0232696

**Published:** 2020-05-06

**Authors:** Rongfeng Zheng, Jiayong Liu, Liang Liu, Shan Liao, Kai Li, Jihong Wei, Li Li, Zhiyi Tian

**Affiliations:** 1 College of Electronics and Information Engineering, Sichuan University, Chengdu, China; 2 College of Cybersecurity, Sichuan University, Chengdu, China; Stevens Institute of Technology, UNITED STATES

## Abstract

The transport layer security (TLS) protocol is widely adopted by apps as well as malware. With the geometric growth of TLS traffic, accurate and efficient detection of malicious TLS flows is becoming an imperative. However, current studies focus on either detection accuracy or detection efficiency, and few studies take into account both indicators. In this paper, we propose a two-layer detection framework composed of a filtering model (FM) and a malware family classification model (MFCM). In the first layer, a new set of TLS handshake features is presented to train the FM, which is devised to filter out a majority of benign TLS flows. For identifying malware families, both TLS handshake features and statistical features are applied to construct the MFCM in the second layer. Comprehensive experiments are conducted to substantiate the high accuracy and efficiency of the proposed two-layer framework. A total of 96.32% of benign TLS flows can be filtered out by the FM with few malicious TLS flows being discarded provided the threshold of the FM is set to 0.01. Moreover, a multiclassifier is selected to construct the MFCM to provide better performance than a set of binary classifiers under the same feature set. In addition, when the ratio of benign and malicious TLS flows is set to 10:1, the detection efficiency of the two-layer framework is 188% faster than that of the single-layer framework, while the average detection accuracy reaches 99.45%.

## Introduction

Plaintext messages can be readily eavesdropped and tampered with during transmission, which poses a great security risk to network users. This plaintext access behavior has been marked as unsafe by Google Chrome. In this context, the transport layer security (TLS) protocol has been widely adopted for its ability to encrypt plaintext and to prevent general man-in-the-middle attacks as mentioned in [1]. According to Sandvine’s latest report [2], encrypted traffic accounts for 50% of global web traffic.

The TLS protocol can guarantee the security of users’ access to the Internet; however, it also facilitates malware to establish command and control (C&C) channels. Malware can briskly pass through the firewall via TLS-based communication technology, and the encrypted payload makes it difficult to analyze. Malicious TLS traffic has also shown an increasing trend in recent years. As portrayed in Cisco’s report in 2018 [3], 33% of malware utilizes the TLS protocol to establish C&C communication. In addition, MITRE ATT&CK [4] has recorded a series of cyber attacks exposed in the past few years, and the number of attacks using 443 ports to establish C&C communication accounts for 66.67%. Therefore, the wide application of the TLS protocol brings a large challenge to achieve the purpose of identifying malicious TLS flows with suitable efficiency.

In industry, the whitelist approach has played an indispensable role in refining malware detection efficiency. Through checking the server name field or domain in the certificate, the TLS flows regarded as “benign” can be filtered out directly. Nevertheless, server names and certificates can be fabricated by malware, which makes the whitelist approach unreliable to some extent.

Facing this sophisticated and untrusted communication environment, this paper proposes a two-layer detection framework with a rapid rate and high precision based on the supervised learning algorithm. Current studies focus on either improving the detection accuracy [5–7] or optimizing the detection efficiency [8, 9]. Few studies discuss how to improve the detection efficiency for a two-layer detection framework without affecting the detection accuracy. Indeed, as long as a majority of benign TLS flows are excluded quickly, both detection indexes can be guaranteed. Moreover, through further exploration of the features of TLS flows, we can establish a more accurate classification model. Accordingly, we propose two models, namely, a filtering model and a malware family classification model. The former is applied to filter out a majority of benign TLS flows, and the latter is employed to identify malware families. The combination of these two models forms our two-layer detection framework. The innovations of this paper are as follows:

1. A binary classifier termed the filtering model based on a new set of TLS handshake features is constructed, in which the accuracy (ACC) and the false positive rate (FPR) can reach 99.82% and 0.072%, respectively. When the threshold of the classifier is set to 0.01, the filtering model can exclude 96.32% of benign TLS flows in advance without affecting the identification of malicious TLS flows.
2. Comparison experiments are conducted between a multiclassifier and a set of binary classifiers under the same feature set to select a better method of dealing with a multiclassification problem. The superior performance of the multiclassifier is verified through comparison experiments.
3. This paper proposed a two-layer framework to refine the efficacy of detecting TLS flows, in which the first layer applies a binary classifier to filter out benign TLS flows and the second layer employs a multiclassifier to identify the malware family of TLS flows. Experiments show that our two-layer framework can greatly improve the detection efficiency, while the detection accuracy is also guaranteed.

The remainder of this paper is arranged as follows. Related work is described in Section 2. A problem statement is introduced in Section 3. Section 4 shows the two-layer detection framework. Section 5 introduces the TLS protocol, especially the TLS handshake information. Section 6 discusses feature engineering, including TLS handshake features, statistical features, and feature selection methods. Section 7 presents the experiments and the related remarks. The conclusion is demonstrated in the last section, in which potential future work is also discussed.

## Related work

For encrypted network traffic, effective identification cannot be done via simply matching signatures used by traditional deep packet inspection (DPI) methods. Because the encrypted payload does not have a fixed string, DPI tools such as Snort [10] do not work. To remedy this drawback, much effort has been devoted to building various detection models via statistical features [11–16], such as the packet size, number of packets and interpacked time.

Some works have focused on discovering and selecting more relevant features among statistical features. A feature selection method utilizing correlation was proposed by Wang et al. [17], in which the least feature set was selected based on KDD Cup 99 dataset [18] and NSL-KDD dataset [19], and high detection efficiency was gained. In a study by McGaughey et al. [20], the fast orthogonal algorithm was applied to select 12 features from 2839 features, which reduced the time overhead by 81% while maintaining the detection rate. Zhang et al. [21] proposed two feature selection algorithms. One is called “WSU_AUC” and is used to deal with the class imbalance problem; the other is termed SRSF and is employed to select robust and stable features. The advancements of the classification model were verified by experiments. Optimizing the feature set can improve the detection efficiency. Nonetheless, in regard to the encrypted network traffic, the classification models based only on the statistical features are insufficient to detect malicious traffic because there exist many false positives that are difficult to analyze.

In the identification of malicious encrypted traffic, some works have also explored other detection methods. Chen et al. [5] designed a multilayer detection framework that was employed to alleviate the class imbalance problem. To improve the detection accuracy, they proposed a tree-shaped deep neural network algorithm along with a quantity-dependent backpropagation algorithm to establish a detection model based on statistical features. Experiments showed that this model could achieve higher detection accuracy than other methods. Comar et al. [6] designed a two-layer detection model and focused on introducing a tree-based feature transformation algorithm to obtain more effective features. The main function of the first layer was also to filter out benign packets, but there was no detailed description of the filtering mechanism, and they did not evaluate whether the method they proposed could improve the detection efficiency. Celik et al. [22] identified malware by heartbeat packets. Zhao et al. [23] detected APT attack traffic by analyzing DNS records. Vadrevu et al. [24] captured malicious flows by identifying download behaviors produced by malware. Bilge et al. [25] used Netflow [26] records in conjunction with an external evaluation system to detect malware C&C communications. However, all these studies depicted above focus on how to refine the detection accuracy and seldom discuss the impact on detection efficiency.

Since the TLS protocol exchanges plaintext information during the handshake phase, more reliable features can be brought to construct the classification model. Cisco engineers Anderson and David et al. have conducted in-depth research on malicious TLS flows. Their main contributions are exploring various new features that can be applied to improve the detection accuracy of TLS flows [27–29]. In [27], the state transition features based on the Markov chain and the byte distribution features are verified by contrast experiments. Context information including DNS responses, HTTP headers, and TLS handshake information are imported to establish classification models of a malware family in [28]. The authors of [29] further discuss TLS handshake characteristics and combine the other 3 kinds of statistical features to detect malicious TLS flows. In the studies mentioned above, the authors all claim that their methods significantly increase the performance of classifiers. However, in the recent study [29], by using only the TLS handshake features, the accuracy of the two-class model was determined to be 98.2%. When the false discovery rate is 0.01%, the accuracy is 63.8%, which means that this method produces many false positives. In the process of reproducing the method of Anderson et al., we found that only a few TLS handshake features are taken into consideration and that TLS handshake features can be further mined. Moreover, there is no detailed discussion on the detection efficiency in these papers. Accordingly, motivated by this prior research, we can train a filtering model by using only TLS handshake features and establish a malware family classification model by utilizing both TLS handshake features and statistical features.

In fact, some researchers are dedicated to improving the detection efficiency of network traffic. Liya et al. [8] used a hierarchical clustering algorithm to divide the samples into multiple clusters. Several representative flows are selected in each cluster. The classification result of these flows is the classification result of the entire cluster by applying the multinomial naive Bayes algorithm. In this way, the detection efficiency can be improved because many flows do not need to be classified. However, a small loss in accuracy does exist in the related experiments. Wang et al. [9] presented the seed expanding (SE) algorithm to optimize clustering performance, which can significantly reduce the number of iterations when two seeds are selected. However, there was no further discussion of the influence of the detection effect. Most of the previous works deal with heavy network traffic via clustering-related methods. There are few discussions on improving efficiency by designing a reasonable detection framework based only on the supervised learning algorithm, and this is exactly what this paper aims to do.

## Problem statement

To detect malicious TLS flows efficiently, this paper proposes a two-layer detection framework. The first layer is designed to filter out benign network traffic; the second layer is utilized to identify malware families of TLS flows. Similar detection frameworks are used in [5] and [6], but in their methods, neither any description of the filtering mechanism nor the efficiency evaluation is mentioned. Simultaneously, the TLS flow is a kind of encrypted network traffic and cannot be filtered by simply matching the signature. For the proposed two-layer detection framework, in addition to the extra consumption time of the filtering model, the traversal times of the two-layer framework are also more than that of the single-layer framework, which may result in the two-layer framework being less efficient than the single-layer framework. To address this disadvantage, the consumption time of the filtering model must be lower than that of the malware family classification model. Accordingly, the first problem is how to train an efficient filtering model (a binary classification model, BC) that can filter out benign TLS flows with a rapid rate and high precision.

Due to the existence of various malicious TLS flows in cyberspace, the malware family classification model focuses mainly on solving a multiclassification problem. To accurately identify the malware family of TLS flows, either a multiclassifier or the “one against all” strategy that utilizes a set of binary classifiers can be applied. However, current studies seldom compare the effects of these two options under the same feature set in the field of network flow detection. Hence, the second problem is which option is better to deal with the multiclassification problem.

## Two-layer detection framework

In a real gigabit network environment, hundreds of TLS flows generated every minute make it costly to identify malware families of TLS flows in real time. In addition, as the number of malware families surges, so does the pressure on the detection system. Hence, it is imperative and appropriate to design a detection framework to reduce the time consumption of TLS flows and guarantee the detection accuracy at the same time.

**Fig 1:**
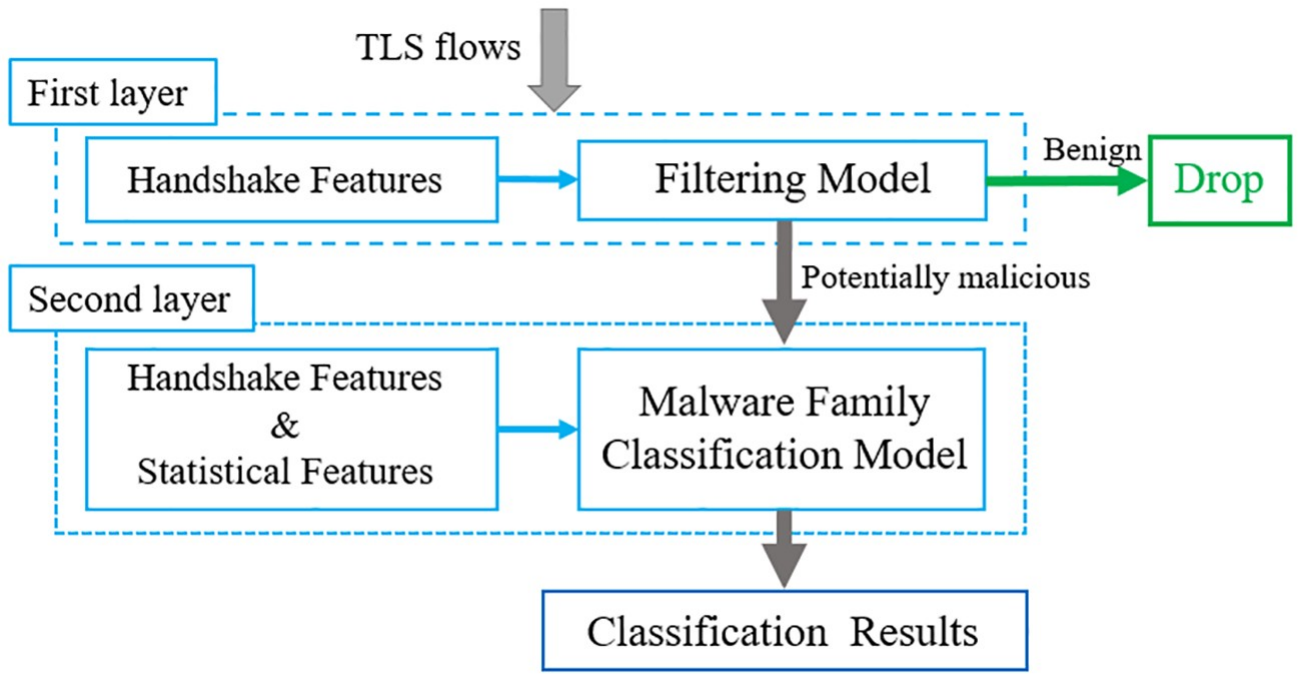
Two-layer detection framework.

We propose a two-layer detection framework as shown in Fig 1. The first layer consists of a BC termed the filtering model, which is applied mainly to filter benign TLS flows based only on TLS handshake features. The second layer is a malware family classification model for identifying the malware family of TLS flows based on both TLS handshake features and statistical features. When a new TLS flow is imported into this detection framework, the detection process is as follows.

The flow is sent to the filtering model; if this model identifies it as a benign TLS flow, it is directly discarded and no longer put into the next layer; if classified as a potentially malicious TLS flow, it passes to the next layer for further identification about which malware family it belongs to. Through this process, one can speculate that the TLS flow that is not discarded by the filtering model may contain both malicious TLS flows and benign TLS flows. However, compared to the number of flows in the first layer, the number of benign TLS flows in the second layer is much less; thus, the detection efficiency can be improved.

To make the two-layer framework more efficient, the time consumed by the first layer must be less than that of the second layer; otherwise, the two-layer framework would reach the opposite destination. In this section, an inequality is used to infer the condition with superior efficiency by the mathematical calculation concerning the time consumed by the two models. A more efficient method will result in lower time overhead. We consider the following inequality:

$$\begin{array}{c} NF*T_{1}+\left(1-r\right)*NF*T_{2}<NF*T_{2} \end{array}$$ (1)

In Eq (1), *NF* represents the number of TLS flows, *T*_1_ represents the average time consumed by the filtering model for every piece of flow, *r* represents the proportion of TLS flows filtered out by the first layer (*r* ∈ [0, 1]), and *T*_2_ represents the average time consumed by the malware family classification model for every flow segment. This inequality can be simplified as follows:

$$\begin{array}{c} r>T_{1}\backslash T_{2} \end{array}$$ (2)

From the inequality, we can conclude that the efficiency of our method depends not on the number of flows but on the proportion of flows filtered out by the first layer. The original range of *r* is [0, 1]. To make the two-layer framework more efficient, the value of *T*_2_ must be greater than *T*_1_. Under this condition, the range of *r* needs to be (*T*_1_\*T*_2_, 1].

Since the model of the first layer has fewer training features than the malware family classification model, the time consumption *T*_1_ of the former is less than the time consumption *T*_2_ of the latter. That is, if the prerequisite condition of In Eq (2) is satisfied, a more efficient detection process can be achieved.

## TLS handshake information

The TLS protocol is derived from the secure sockets layer (SSL) protocol. The TLS protocol version has been updated to 1.3, but the mainstream version is still 1.2. Few apps implement version 1.3, which regulates the samples collected in this paper to be based mainly on TLS 1.0, TLS 1.1 and TLS 1.2.

**Fig 2:**
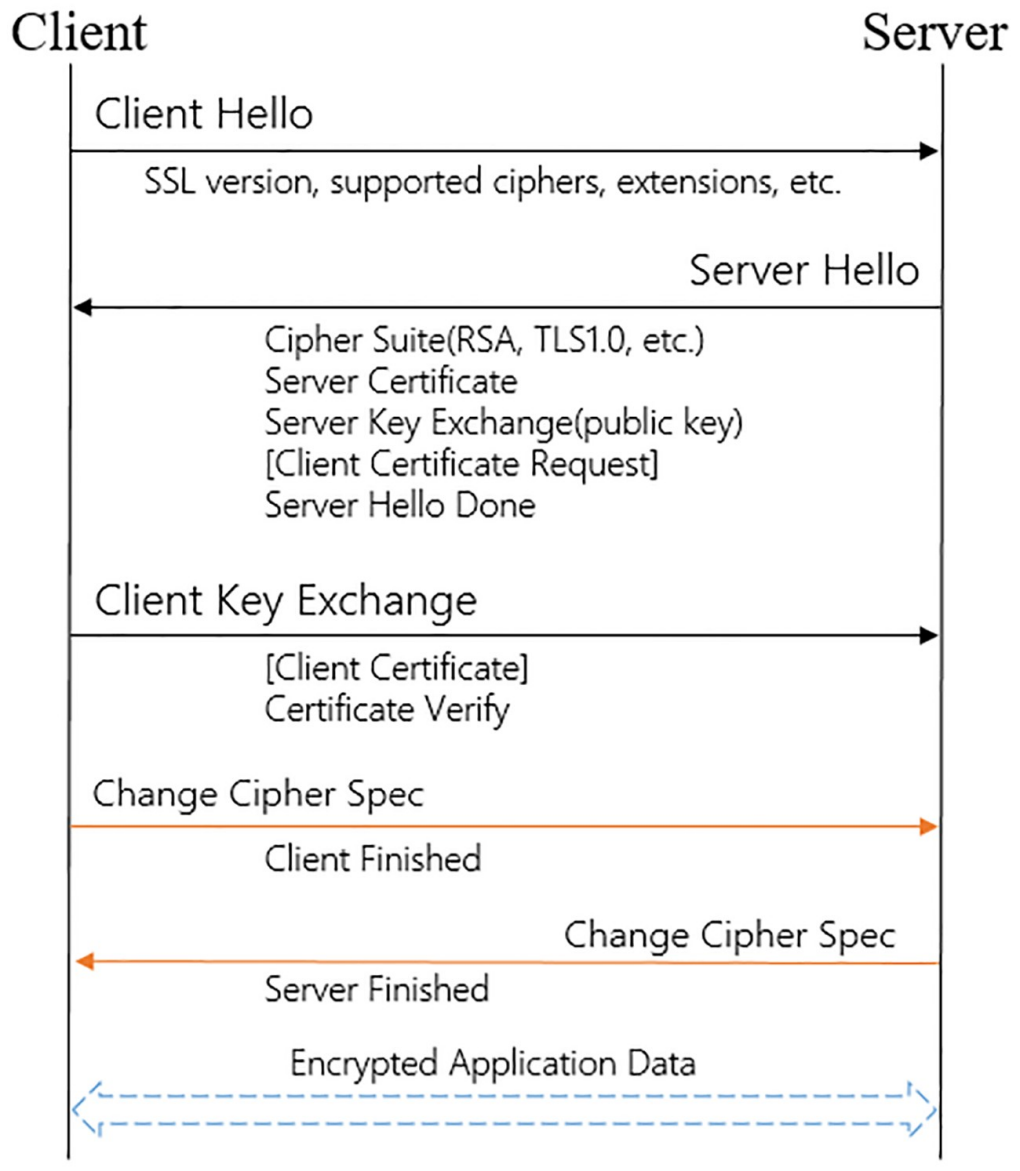
TLS protocol key negotiation process.

Fig 2 shows a typical process for TLS key negotiation. In this process, two main purposes are completed, namely, key negotiation and identity authentication, and the message information exchanged between the client and the server is the focus in this paper. In the figure, the client hello contains the TLS version, cipher suites, extensions, etc. The server hello includes the TLS version, cipher suite, extensions, certificate, server key exchange, and client certificate request. In the change cipher specification, since the message between the client and server is very constrained, this paper does not consider extracting features from it. In fact, much plaintext information is exchanged in the key negotiation phase except for a few encryption fields.

Because the negotiation information generated by different software programs is not completely the same, such as the cipher suites, server name, and certificate information, it is not feasible to extract the signature features that can be used to identify the TLS flows. Different applications may also adopt the same cipher suite and other negotiation information.

To save computing resources of the server, malware tends to adopt simple encryption algorithms and provides little handshake information [28], which allows benign applications and malware to show many differences during the key negotiation phase.

## Feature engineering

### TLS handshake feature

In Anderson et al.’s method [29], three main types of features are used: the list of offered cipher suites, the list of advertised extensions, and the public key length. A total of 198 TLS handshake features are selected in their method. However, in the TLS key negotiation phase, there are differences not only in these fields but also in other fields, such as the protocol version, server name, client hello length (CHL), cipher suite number, client/server extension number, and certificate number. This paper compares the discrimination between benign and malicious samples in these fields (refer to the discussion of data collection for details about the sample set).

Protocol version: The protocol version used by most of the benign applications is TLS 1.2, and the TLS flows with lower protocol version account for only 2.19% of the entire TLS flows. However, among malicious TLS flows, the proportion of the lower protocol version is higher, reaching 30.28%.

Server name: There are different forms in the representation of this field. This field may be empty, filled with the domain generation algorithm (GDA) domain, or filled with IP addresses. The corresponding proportions are 0.51%, 17.77%, and 1.32% in the benign TLS flows; however, in malicious TLS flows, these proportions are 71.36%, 4.0%, and 0%.

**Fig 3:**
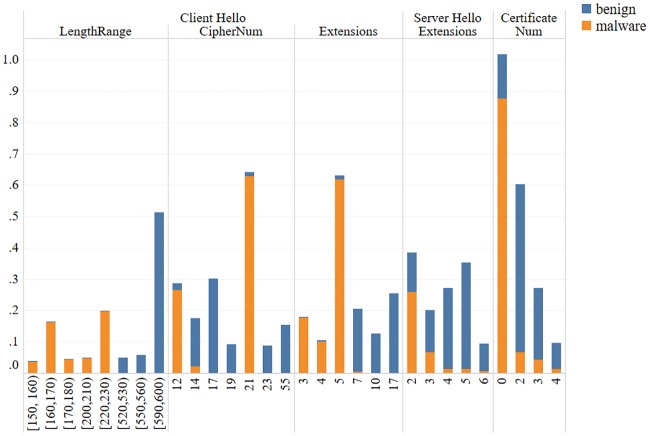
Differences in other fields.

Other fields: Since these fields are all represented by numerical values, we group them for convenience of description. In general, malware has smaller values in these fields, while benign TLS flows tend to have a longer CHL and a larger cipher suite number, extension number, and certificate number. From these fields, some features are selected to draw Fig 3 according to 2 criteria: the value of each feature is larger than 0.05, and the ratio of benign to malicious (or malicious to benign) samples at each feature is larger than 3. The features that satisfy both criteria can be selected in Fig 3. A trend is observed that as the numerical value is larger, the proportion of malicious TLS flows is lower, while the proportion of benign TLS flows is higher.

In addition, sparse representation is applied to design the features of each field. For example, under the client hello version field, we set 3 features, namely, TLS 1.0, TLS 1.1 and TLS 1.2. Under the CHL field, we set 150 bins of 10 bytes each, from [0, 10) to [1490, 1500); if the CHL is greater than 1490, it is put into the last bin. Each bin represents a feature, so there are a total of 150 features in the CHL field. The value of CHL belongs to a certain bin; this bin’s value is 1, and the remaining values are 0. These features are called “sparse features”, and we use them to devise our feature set. In addition to the features proposed by Anderson et al. [29], we further mine the other 6 kinds of TLS features, including the client/server hello version, CHL, cipher suite number, client/server extension number, server name, and certificate number. As shown in Table 1, there are a total of 705 features.

**Table 1:** TLS handshake feature set.

Feature Name	Description	Feature Number
Client hello version (*new*)	Which version it belongs to	3
CHL (*new*)	Which bin it belongs to (10 bytes per bin)	150
Cipher suite number (*new*)	Which number it belongs to	128
Client cipher suites	Which cipher suites it belongs to	190
Client extension type	Which extension type it belongs to	32
Client extension number (*new*)	How many extensions it has	32
Server name (*partly new*)	If it is in the top 1 million DNS Alexa (*not new*), empty, random string or IP	4
Client public key length	Which key length it belongs to	12
Client signature algorithm number	Which number it belongs to	9
Client padding length	Which bin it belongs to (8 bytes per bin)	32
Server hello version (*new*)	Which version it belongs to	3
Server cipher suite	Which cipher suite it belongs to	30
Server extensions type	Which extensions type it belongs to	32
Server extension number (*new*)	How many extensions it has	32
Certificate number (*new*)	How many certificates it has	16

### Statistical features

To accurately identify malware families of TLS flows, it is insufficient to use just the TLS handshake features. Anderson et al. [29] demonstrated that TLS handshake features combined with statistical features can achieve higher detection accuracy than other techniques in identifying malware families. Here, we refer to the research of predecessors and select a set of statistical features that have been verified. Aksoy et al. [30] utilized the features in packet headers to train classifiers. The validity of the packet length distribution and time interval distribution is demonstrated in [31]. The first packet length and minimum packet length feature are used in [32]. The Markov chain generated by the sequence of the length and time interval among packets is mentioned in [33], and the state transition probability is used as the feature. By taking advantage of the research results of predecessors, as shown in Table 2, we summarize the statistical features in this paper.

**Table 2:** Statistical features.

Description	Feature number
Min. packet length	2
Max. packet length	2
First packet length	2
Packets with a push flag	2
Packet length distribution	150
Packet interarrival time distribution	100
Byte distribution	256
Packet interarrival time transition probability matrix	100
Packet length transition probability matrix	100

In Table 2, since we take the direction of the flow into account (client to server and server to client), the min. packet length is represented by two features, the same as the max. packet length, first packet length, and packets with a push flag. For the packet length distribution, we also set 150 bins of 10 bytes each and calculate the length distribution of the first 100 packets among the 150 bins. For the packet interarrival time distribution, we set 100 bins of 5 ms each, and any interarrival time of more than 495 ms is put in the last bin. Then, we calculate the interarrival time distribution of the first 100 packets among the 100 bins. For the byte distribution, we compute the ratio of each byte count to the total number of bytes in the packet payload. There are 256 representations of a byte, so there are 256 features. For the packet interarrival time transition probability matrix, we set 10 bins of 50 ms each, and any interarrival time of more than 450 ms is put in the last bin. We calculate the transition probability matrix with the first 100 packets based on the Markov chain. Similarly, for the packet length transition probability matrix, we set 10 bins of 150 bytes each and calculate the length transition probability matrix by utilizing the first 100 packets. The statistical features are combined with the handshake features to establish a more accurate classification model for identifying malware families.

### Feature selection

Because we use sparse representation to design our feature set, the produced features are high dimensional. Inevitably, there are some irrelevant features in the feature set. For this reason, before training the model, we need to reduce the number of feature dimensions by removing those irrelevant features. Because the filtering method does not depend on a specific machine learning method, it has the characteristics of high operational efficiency and is suitable for solving the problem of feature selection in high-dimensional data. We use the information gain [34], which is one of the filtering methods, to select more relevant features. The information gain can be expressed as the difference between the entropy and conditional entropy, as shown in the following equation:

$$\begin{array}{c} IG(X)=H(C)-H(C|X) \end{array}$$ (3)

In Eq (3), *H*(*C*) stands for the information entropy, and its essence is the measure of the uncertainty of random variables. Its definition is as follows:

$$\begin{array}{c} H\left(C\right)=-\sum_{i=1}^{n}\;P\left(C_{i}\right)\log_{2}\;P\left(C_{i}\right) \end{array}$$ (4)

In Eq (3), *H*(*C* ∣ *X*) stands for the conditional entropy, which is a measure of the uncertainty of random variable *c* with a certain value of *x*. Its definition can be seen in Eq (5):

$$\begin{array}{c} H\left(C|X\right)=\sum_{x\in X}\;p\left(x\right)\;H\left(C|X=x\right) \end{array}$$ (5)

From the above three formulas, the information gain of each feature can be conveniently computed. By comparing the information gain of each feature, the importance of features can be measured, and by filtering out the features with low information gain, the feature dimension can be reduced.

## Experiments and results

To demonstrate the effectiveness of our methods, comprehensive experiments are conducted. There are mainly 4 parts: 1) Detailed methods of collecting samples are presented in the data collection part. 2) The filtering model is established and evaluated through the selection of relevant features and a reasonable threshold. 3) A multiclassifier and a set of binary classifiers are compared to select a better method for dealing with the multiclassification problem in the evaluation malware family classification model. 4) The two-layer detection framework is evaluated by comparing it with the single-layer framework.

### Data collection

In this section, the collection methods of the sample set and the necessary preprocessing steps are presented in detail. The Streamdump tool (https://github.com/NewBee119/StreamDump) we developed is used to collect TLS flows according to the quad information {srcIP, srcPort, dstIP, dstPort}. There are two ways for StreamDump to reassemble TLS packets. One is monitoring network traffic on a network adapter, where the transport layer protocol is TCP and the destination port is 443. Another is directly reading .pcap files that are saved by others. During data collection, both methods are used to collect TLS flows. For collecting benign TLS flows, StreamDump is used to reassemble real-time TLS packets, but for malicious samples that are shared by others in the form of .pcap files, StreamDump is utilized to extract malicious TLS flows from these files. Moreover, the handshake type field is applied to determine whether a TLS flow contains a complete handshake process, and TLS flow samples that do not contain the complete handshake process are discarded.

For the collection of benign TLS flow samples, we spent 15 days collecting a total of 1323667 TLS flows from our laboratory network. Before using these samples, we need to conduct several preprocessing steps on these samples. First, there are many TLS flows without the entire TLS handshake process because of some optimization schemes, such as session tickets. However, when the connection to the server occurs for the first time or when the session ticket time runs out, the entire TLS handshake process is required to connect to the server. Therefore, we need to exclude the flows that do not contain the entire TLS handshake information, and 590093 flows remain. In addition, to objectively reflect the differences between benign and malicious TLSs, we delete the TLS flows that have the same server name and CHL from these samples and obtain 21743 flows after this step. In fact, at this point, we still cannot guarantee that the TLS flows obtained in the previous steps are all benign, and further preprocessing is needed. This paper uses the open-source threat community AlienVault to check whether the destination IP of a TLS flow is potentially malicious. We developed the check_ip tool (https://github.com/NewBee119/check_ip) by using AlienVault’s API to discover and filter out the potential malicious TLS flows, which can ensure the purity of benign samples. Through preprocessing, we selected 18241 benign TLS flows from 1323667 TLS flows, which not only improves the quality of the sample set but also alleviates the problem of class imbalance to some extent.

For the collection of malicious TLS flow samples, we collect malware traffic samples shared on the Internet by using our own crawler tool (https://github.com/NewBee119/malware_traffic_crawler). Malware Traffic [35] is a website which shares malicious network traffic and malware samples since 2013. We only focus on .pcap files which can be used to extract TLS flows by the StreamDump, and malware samples should not be run on your own machine in case of being infected. We have obtained 15077 TLS flow samples in Malware Traffic. Another source is the BCIC dataset [36] which also shares malicious traffic and malware samples, and a total of 210,484 TLS flows are extracted. These flows are generated in the virtual machine by executing malware. However, there is a problem that we cannot tell whether the TLS flows are generated by malware or by other, benign applications in the virtual machine. To improve the reliability of the training data, we still use AlienVault’s API to filter out TLS flows that are identified as benign. After these steps, we finally obtain 17923 malicious TLS flows that have a complete handshake phase. Accordingly, we select some malware families to verify our method, and malware families with less than 100 flows are not selected. The number of TLS flows for each malware family is shown in Table 3.

**Table 3:** TLS flows in each malware family.

Malware family	Number of flows	Unique server IPs
EITest	135	53
Emotet	1898	144
Hancitor	2613	80
Nuclear	262	19
Rig	245	49
Trickbot	1600	115
Dridex	5074	12
Razy	1019	1
HTBot	695	19

### Evaluation of the filtering model

The filtering model as a coarse classification model is employed mainly to quickly filter out the benign TLS flows and to ensure that the malicious TLS flows are passed to the next layer as much as possible. Three steps are presented to reach this goal: 1) selecting the relevant TLS handshake features; 2) verifying the effectiveness of the filtering model; and 3) selecting the appropriate threshold for the filtering model.

Since we obtain 705 TLS handshake features, the feature dimension needs to be reduced before the training model. Based on the information gain algorithm mentioned in the previous section, we can calculate the information gain value (IGV) for each feature and select candidate feature sets based on the IGV. The detailed process is presented in Algorithm 1. The modified wrapper method with a backward selection strategy is used to select the best feature subset. The information gain of each feature should be calculated in advance. *IG*(*F*_*i*_) represents the result of information gain for feature subset *F*_*i*_, *T*_*i*_ represents the *i*th feature subset, and *F*_0_ represents the original feature set. The ACC and FPR can be calculated by the classifier. *X*_*labeled*_ represents the labeled benign samples and malicious samples.

**Algorithm 1** Modified wrapper method for feature selection

**Require**: *F*_0_, *IG*(*F*_0_), *X*_*labeled*_

**Ensure**: the best feature subset (*BFS*)

1: select the classifier based on the logistic regression algorithm;

2: based on IG(*F*_0_), sort(*F*_0_) in descending order, and obtain the sorted *F*_0_’;

3: calculate $ACC_{F_{0}}$ and $FPR_{F_{0}}$ for *X*_*labeled*_;

4: **for** backward select $F_{0}^{\prime }$, and obtain *F*_*i*_ **do**

5:  **if** min(*IG*(*F*_*i*_)) is equal to 0 **then**

6:   continue;

7:  **end if**

8:  calculate $ACC_{F_{0}}$ and $FPR_{F_{0}}$ for *X*_*labeled*_;

9:  **if** $ACC_{F_{i}}$ < $ACC_{F_{i-1}}$ and $FPR_{F_{i}}$ > $FPR_{F_{i-1}}$ **then**

10:   $ACC_{F_{i}}$ = $ACC_{F_{i-1}}$, $FPR_{F_{i}}$ = $FPR_{F_{i-1}}$;

11:   **if** *BFS* is *NULL* **then**

12:    *BFS* = *F*_*i*−1_;

13:   **end if**

14:  **else** {$ACC_{F_{i}}$ ≥ $ACC_{F_{i-1}}$ or $FPR_{F_{i}}$ ≤ $FPR_{F_{i-1}}$}

15:   *BFS* = *F*_*i*_;

16:  **end if**

17: **end for**

18: **return** *BFS*

There are three main steps in Algorithm 1: 1) preparatory work (lines 1-2); 2) calculating the initial parameters based on classifier (line 3); 3) evaluating *F*_*i*_ and selecting the best feature subset (lines 4-17). In step 3, the backward selection strategy is used to construct a feature subset (*F*_*i*_), and the number of features in *F*_*i*_ is 1 less than that in *F*_*i*-1_. The features in which the IGV is 0 can be directly excluded because they have no contribution to the classifier (lines 5-7). Different from the original wrapper method, our proposed Algorithm 1 can skip irrelevant features and screen out feature subsets with the highest detection accuracy. The feature subset that can achieve the highest ACC can be regarded as the best feature subset (lines 8-16).

**Fig 4:**
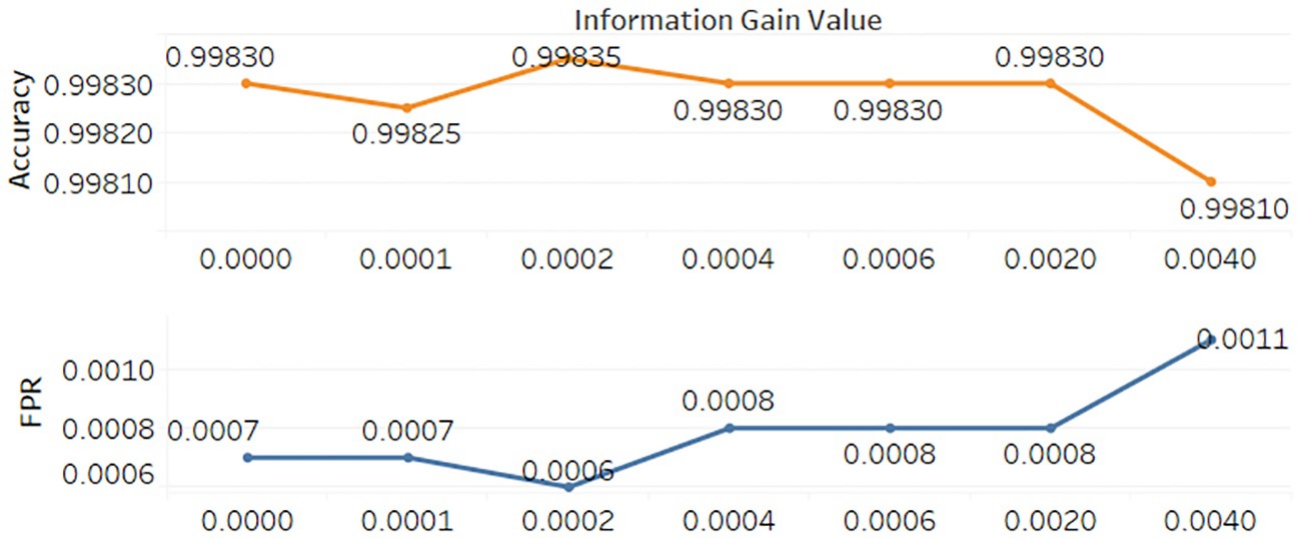
Classification results among different feature sets.

To alleviate the class imbalance problem [37], we randomly select 10,000 benign samples and 10,000 malicious samples and utilize the logistic regression algorithm to evaluate the performance among different feature subsets by calculating the ACC and FPR. As shown in Fig 4, we select 7 feature subsets to exhibit the process described in Algorithm 1, from the feature subset in which the minimum IGV is equal to or greater than 0 to the feature subset in which the minimum IGV is equal to or greater than 0.004.

Fig 4 shows the feature subset in which the minimum IGV is equal to or greater than 0.0002, from which we can obtain the best classification results in which the ACC is the highest and the FPR is the lowest compared with other feature subsets. Under this condition, 297 effective features can be screened out and used to train our filtering model.

For comparison, we completely reproduce Anderson et al.’s method [29] by utilizing the logistic regression algorithm to train classifiers and 10-fold cross-validation to evaluate the performance. The features used in our method include the 6 kinds of features we proposed; the features without a *new* tag in Table 1 are used by Anderson et al.’s method. The ACC and FPR are calculated by adopting their method and our method among different numbers of TLS flow samples.

**Fig 5:**
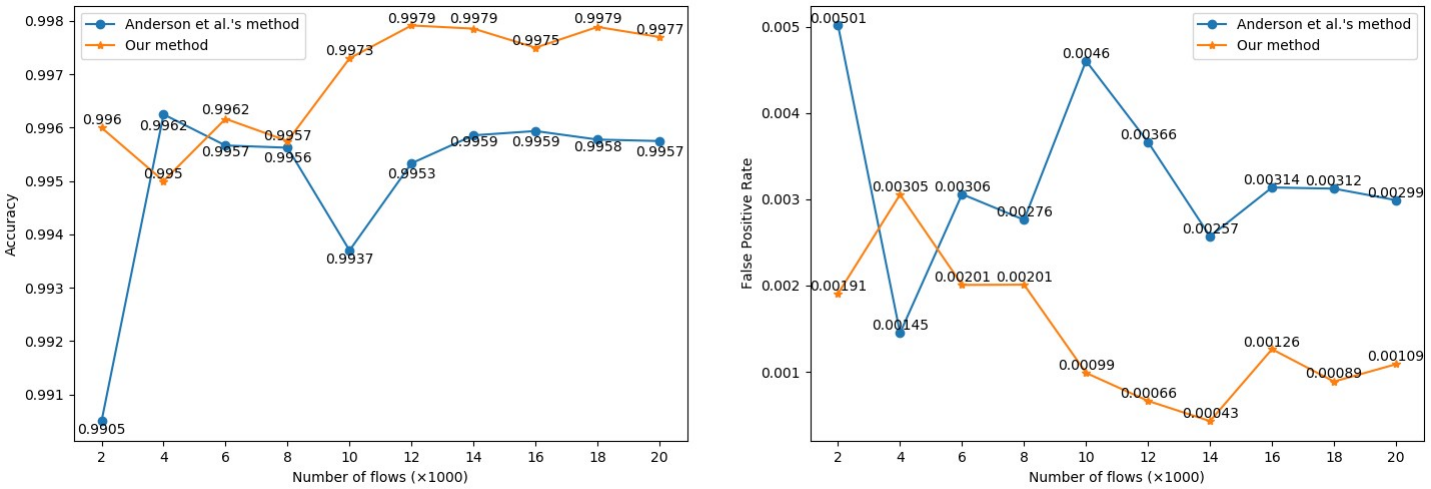
Comparison of the two methods.

Fig 5 shows the comparison results of the two methods with the sample number ranging from 2,000 to 20,000. The ratio of positive and negative samples is 1:1. When the sample size increases past 10,000, both the ACC and FPR become gradually stable, and we can calculate the average ACC and average FPR under this condition. By applying the 6 kinds of features we newly proposed, the average ACC of our classifier is 99.78%, and the average FPR is 0.09%. Compared with Anderson et al.’s method [29], the average ACC of our method is 0.20% higher than that of their method, while the average FPR is 0.22% lower than their method. Since the average ACC of Anderson et al.’s method is very high, reaching 99.58%, the 0.2% improvement is also considerable. Therefore, by further mining TLS handshake features, we can establish a better BC than Anderson et al.’s method [29].

**Fig 6:**
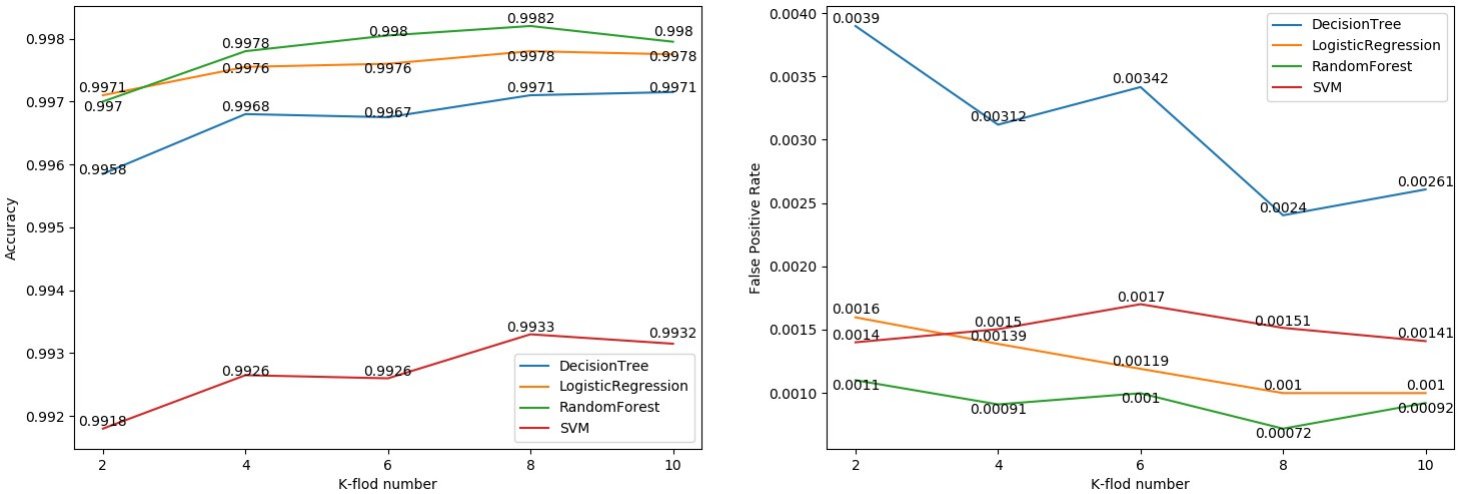
Comparison among the 4 different algorithms.

Moreover, we also compare the classification effects among different machine learning algorithms. A total of 20,000 samples with the same number of benign and malicious TLS flows are used to calculate the ACC and FPR under *k*-fold cross-validation. As shown in Fig 6, all 4 algorithms can achieve a high ACC, but the performance of the random forest algorithm is the best both in the ACC and in the FPR, with the ACC being 99.82% and the FPR being 0.072%. Therefore, we select the random forest algorithm to train our filtering model.

The contribution of features can also be evaluated by the classifier based on the random forest algorithm. The 20 most important features are shown in Table 4. The cipher suites occupy nearly half, which means that the client cipher suites used by benign applications and malware are remarkably different since malware is tends to utilize simpler algorithms to encrypt network traffic. There are 7 features we newly propose with a *new* tag in this paper, which demonstrates the effectiveness of the features we proposed.

**Table 4:** The 20 most important features in the filtering model.

Feature description	Importance
Client cipher suites: TLS_RSA_WITH_RC4_128_MD5	0.0920
Client cipher suites: TLS_DHE_DSS_WITH_3DES_EDE_CBC_SHA	0.0716
Client extension type: extended master secret	0.0557
Client cipher suites: TLS_ECDHE_RSA_WITH_AES_256_GCM_SHA384	0.0539
Client signature number: 2 (*new*)	0.0435
Client cipher suites: TLS_ECDHE_RSA_WITH_CHACHA20_POLY1305_SHA256	0.0390
Client cipher suites: TLS_DHE_DSS_WITH_AES_256_CBC_SHA	0.0354
Client cipher suites: TLS_RSA_WITH_AES_256_GCM_SHA384	0.0318
Client extension type: application layer protocol negotiation	0.0296
Client cipher suites: TLS_ECDHE_RSA_WITH_AES_128_GCM_SHA256	0.0242
Server name is not in the top 1 million DNS Alexa	0.0234
Client cipher suites: TLS_ECDHE_ECDSA_WITH_CHACHA20_POLY1305_SHA256	0.0232
Server name is a random string (*new*)	0.0225
Client extension type: session ticket	0.0195
Server extension number: 1 (*new*)	0.0172
Client cipher suites: TLS_RSA_WITH_RC4_128_SHA	0.0164
CHL: [150, 160) (*new*)	0.0163
CHL: [610, 620) (*new*)	0.0156
Server name is empty (*new*)	0.0150
Client extension number: 5 (*new*)	0.0142

The main function of the filtering model is to filter out benign traffic, while all malicious TLS flows need to be left. We can reach this goal by setting a reasonable decision threshold in the filtering model and use all the testing samples, including 18241 benign samples and 17923 malicious samples, to evaluate our classifier. We used 10-fold cross-validation and the random forest algorithm to calculate the confusion matrix for each threshold.

In Table 5, when the threshold is set to 0.01, the value of FN is 0, which means that all malicious TLS flows can be identified as malicious. On the other hand, the value of TN is 17812, which means that 17812 TLS flows are not passed to the second layer because they are regarded as benign, and these TLS flows account for 97.65% of the total benign TLS flows. Thus, by adopting the random forest algorithm and setting the threshold to 0.01, we can establish our filtering model based only on TLS handshake features.

**Table 5:** Confusion matrix among different thresholds.

Threshold	Confusion matrix			
	TP	FN	TN	FP
0.4	17909	14	18235	6
0.1	17914	9	18172	69
0.05	17928	5	18101	140
0.01	17923	0	17812	429

### Evaluation of the malware family classification model

Generally, identifying malware families of TLS flows is a multiclassification problem. To deal with this problem, there are two options to select. The first option is to train a multiclassification model (MC); the second is using the one against all strategy by training a set of binary classification models (BCs), and each model corresponds to a kind of malware family. Experiments are designed to explore which of the two options performs better.

We prepare 9 kinds of malware families and 18241 benign TLS flows, as shown in Table 3. For the first option, we only need to train a multiclassifier; for the second option, we train 10 binary classifiers in advance (9 for malware families, 1 for benign samples) and select the highest probability among 10 binary classifiers as the classification result during the test.

**Fig 7:**
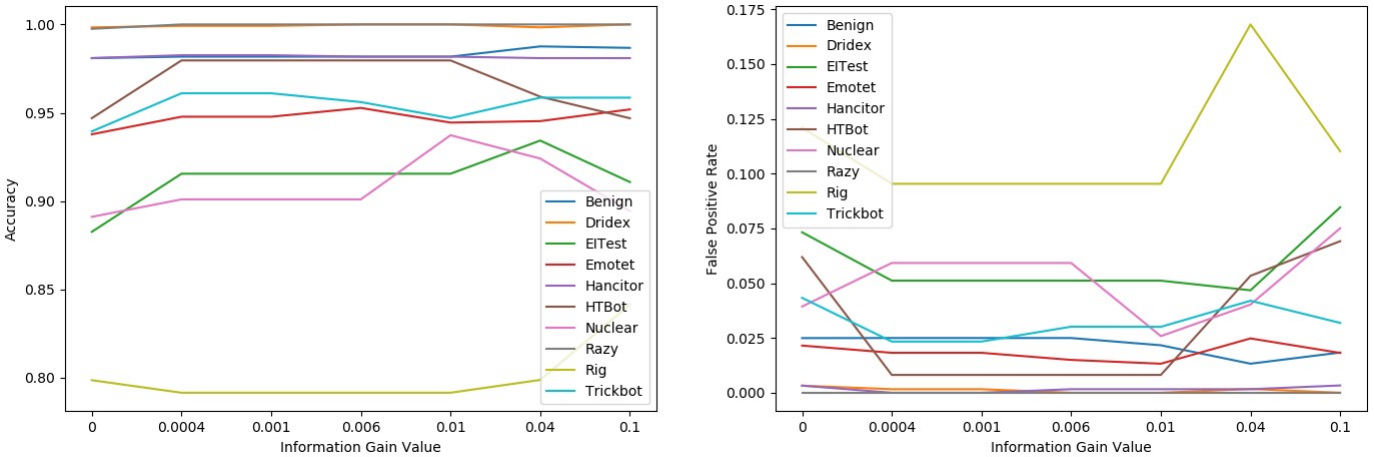
Classification results from 10 binary classifiers among different feature set.

Before training the models, it is necessary to select relevant features from the original 705 TLS handshake features and 664 statistical features. Nevertheless, the information gain algorithm cannot be directly used to select features for a multiclass sample set. The feature selection method we used here contains two steps: 1) selecting relevant features for each binary classifier by utilizing the information gain algorithm and 2) utilizing the union of 10 feature sets selected from 10 binary classifiers as our feature set. The process of feature selection for each binary classifier is the same as that in the filtering model. As shown in Fig 7, the ACC and FPR of each binary classifier are calculated among different feature sets.

After completing these two steps, we finally obtain 762 features, including 234 TLS handshake features and 528 statistical features, and use the random forest algorithm to train our binary and multiple classifiers. We also use 10-fold cross-validation to evaluate the performance of these two options. As shown in Table 6, the performance index of these two options are demonstrated.

**Table 6:** Comparison of the two options.

Malware family	MC			BCs		
	Precision	Recall	F1-score	Precision	Recall	F1-score
Dridex	100%	100%	100%	100%	100%	100%
EITest	97.84%	83.81%	90.25%	97.64%	80.00%	87.90%
Emotet	98.28%	94.37%	96.28%	98.49%	94.01%	96.20%
Hancitor	99.56%	99.62%	99.59%	99.49%	99.65%	99.57%
HTBot	100%	81.74%	89.53%	100%	84.35%	91.27%
Nuclear	98.52%	90.00%	94.02%	97.78%	86.00%	91.45%
Razy	100%	100%	100%	100%	100%	100%
Rig	82.60%	64.62%	72.13%	93.06%	58.46%	71.45%
Trickbot	92.23%	94.06%	93.13%	91.31%	94.39%	92.82%
Benign samples	98.62%	99.99%	99.30%	98.63%	100%	99.31%
**Average ACC**	98.41%			98.36%		
**Time consumption (s)**	108.62			232.51		

The overall performance of the MC is slightly better than that of the BCs, and their average accuracies are 98.41% and 98.36%, respectively. However, due to the mechanism of the second option, which is required to traverse all binary classifiers before obtaining the classification result, the time consumption difference between the two options is remarkably conspicuous, as the time consumption of the BCs is twice that of the MC. Accounting for the superiorities in accuracy and efficiency, we adopt the first option (multiclassifier) to identify the malware family of TLS flows. In a multiclassifier, the importance of each feature can also be evaluated, and the 20 most important features are presented in Table 7.

**Table 7:** The most important 20 features in the malware family classification model.

Feature description	Importance
Client cipher suites: TLS_ECDHE_RSA_WITH_AES_128_GCM_SHA256	0.0481
Client cipher suites: TLS_ECDHE_RSA_WITH_AES_256_GCM_SHA384	0.0272
Client cipher suites: TLS_ECDHE_RSA_WITH_CHACHA20_POLY1305_SHA256	0.0262
Client cipher suites: TLS_RSA_WITH_RC4_128_MD5	0.0250
Cipher suite number: 21 (*new*)	0.0249
Certificate number: 1 (*new*)	0.0236
Server extension number: 1 (*new*)	0.0228
Client cipher suites: TLS_DHE_DSS_WITH_AES_256_CBC_SHA	0.0189
Client cipher suites: TLS_RSA_WITH_RC4_128_SHA	0.0183
Client signature number: 2 (*new*)	0.0162
Server name is not in the top 1 million DNS Alexa results	0.0138
Server cipher suite: TLS_RSA_WITH_AES_128_CBC_SHA256	0.0130
Packet length distribution: [1490, 1500) (*Statistical features*)	0.0124
Server name is a random string (*new*)	0.0117
Packet length distribution: [180, 190) (*Statistical features*)	0.0116
Client extension number: 5 (*new*)	0.0114
Client cipher suites: TLS_ECDHE_ECDSA_WITH_CHACHA20_POLY1305_SHA256	0.0113
Packet interarrival time transition probability matrix: [100, 150 ms) (*Statistical features*)	0.0108
Packet length transition probability matrix: [980, 990) (*Statistical features*)	0.0101
Client extension number: 3 (*new*)	0.0091

From Table 7, there are 7 features related to client cipher suites, which means that different malware families are intended to select different such suites. There are still 7 features we newly propose with a *new* tag in this paper, thus demonstrating again the effectiveness of the features we propose. Moreover, TLS handshake features occupy a majority compared to statistical features (that is, 16:4), so we can conclude that the TLS handshake features are more important than statistical features.

### Evaluation of the two-layer detection framework

**Fig 8:**
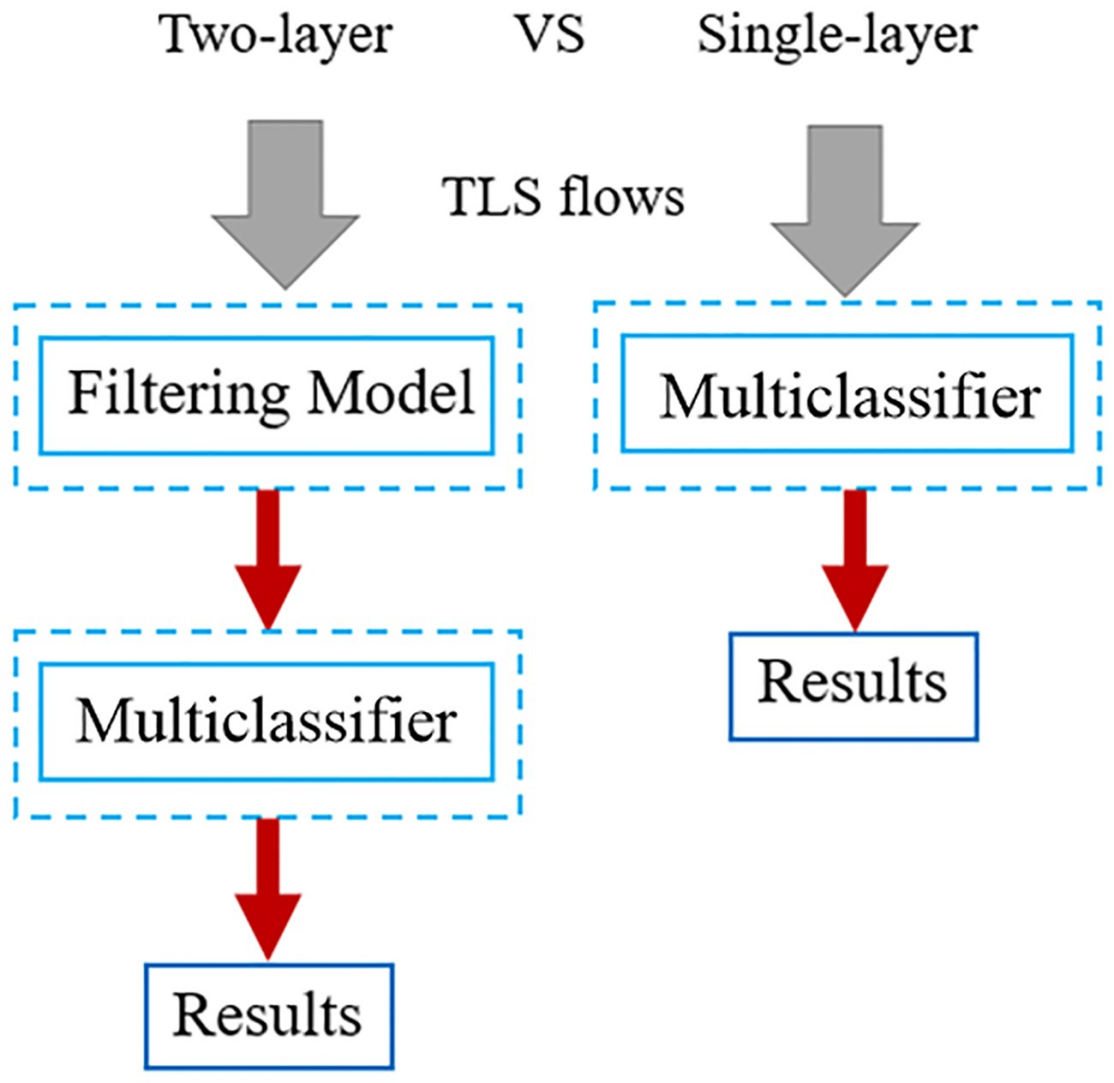
The processing flow of the two frameworks.

In previous experiments, we trained the filtering model (a binary classifier) and the malware family classification model (a multiclassifier). Combining these two models constitutes our two-layer detection framework. To verify the efficiencies of the two-layer framework, contrast experiments between it and a single-layer framework are conducted. As shown in Fig 8, the multiclassifier used in the single-layer framework is the same as the classifier utilized in the second layer of the two-layer framework. The purpose is to evaluate whether the two-layer framework can improve the detection efficiency on the one hand and guarantee the detection accuracy on the other hand.

Since benign TLS flows generally account for the majority of flows in a real network environment, it is reasonable to set the number of benign samples to be greater than the number of malicious samples. We prepared a total of 11,000 TLS flows for contrast experiments, including 10,000 new benign samples and 1,000 malicious samples. Before the experiment, we set the threshold of the filtering model to 0.01, as discussed in the filtering model. Moreover, we adopt the random forest algorithm to train both the filtering model and the multiclassifier in advance. By importing the testing samples into these two detection frameworks, we compare the relative indicators as shown in Table 8.

**Table 8:** Comparison of the two frameworks.

Malware family	Single-layer			Two-layer		
	Precision	Recall	F1-score	Precision	Recall	F1-score
Dridex	100%	100%	100%	100%	100%	100%
EITest	100%	80.00%	88.89%	100%	80.00%	88.89%
Emotet	93.48%	93.99%	93.73%	93.48%	93.99%	93.73%
Hancitor	98.59%	99.29%	98.94%	98.59%	99.29%	98.94%
HTBot	100%	76.92%	86.96%	100%	76.92%	86.96%
Nuclear	100%	82.00%	90.11%	100%	82.00%	90.11%
Razy	100%	100%	100%	100%	100%	100%
Rig	92.31%	54.55%	68.57%	92.31%	54.55%	68.57%
Trickbot	87.92%	95.62%	91.61%	87.41%	91.24%	89.29%
Benign samples	99.81%	100%	99.91%	99.75%	100%	99.88%
**Average ACC**	99.51%			99.45%		
**Traversal Times**	11000			11377		
**Time consumption (ms)**	1360.84			722.44		

Table 8 shows that the two-layer framework does not significantly affect the detection results of TLS flows. Although the traversal times of the two-layer framework are larger than that of the single-layer framework, the time consumption decreases by 188% compared to that of the single-layer framework, which means that the efficiency improves by 188%. Meanwhile, the average detection accuracy of the two-layer framework reaches 99.45%, which produces only a 0.06% loss, which means that the proposed framework also guarantees the detection accuracy. In fact, in the process of the experiment, there are 9623 TLS flows filtered out by the filtering model, which accounts for 96.32% of benign samples, and 87.56% of all test samples. At the same time, few malicious TLS flows are filtered out, which proves the reliability of the filtering model. Moreover, we can compute the average time consumption of each flow in the filtering model and the multiclassifier, respectively, as 0.06 ms and 0.12 ms. Substituting these calculated parameters into In Eq (2), where *r* is 87.56%, *T*_1_ is 0.06 ms and *T*_2_ is 0.12 ms, the accuracy of In Eq (2) is substantiated.

**Fig 9:**
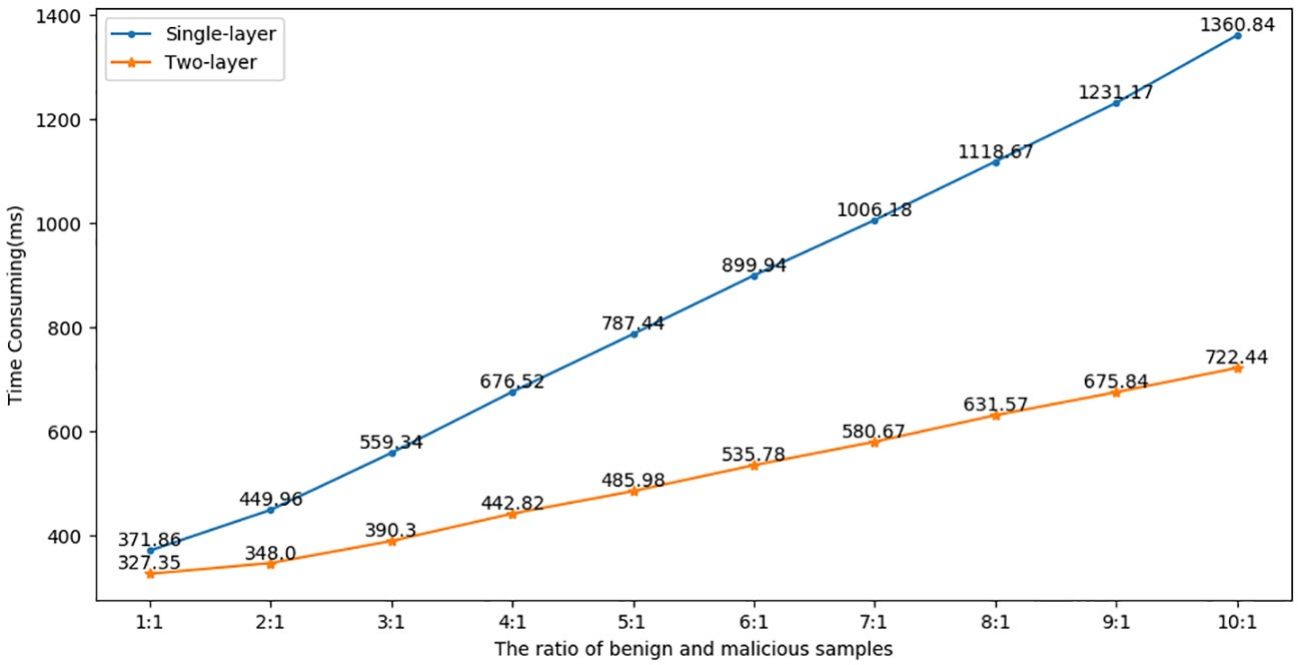
Time consumption at different ratios.

We also compare the time consumption at different ratios of benign and malicious samples. At each ratio, we test a total of 10 times and calculate the average time consumption. As shown in Fig 9, when the ratio is 1:1, the single-layer framework is not much different from the two-layer framework. However, along with the increase in the number of benign samples, the two-layer framework is increasingly advantageous. When the ratio reaches 10:1, the two-layer framework is nearly twice as fast as the single-layer framework. In the real network environment, since benign TLS flows account for the vast majority (the ratio is far more than 10:1), application of the two-layer detection framework is well justified.

In summary, we demonstrate that the two-layer detection framework needs to meet certain conditions to improve the detection efficiency of TLS flows. That is, 1) the detection efficiency of the coarse classification model in the first layer must be higher than that of the detection models in the second layer; 2) the ratio of flows filtered by the first layer must satisfy In Eq (2). Otherwise, the improvement in detection efficiency cannot be guaranteed.

**Fig 10:**
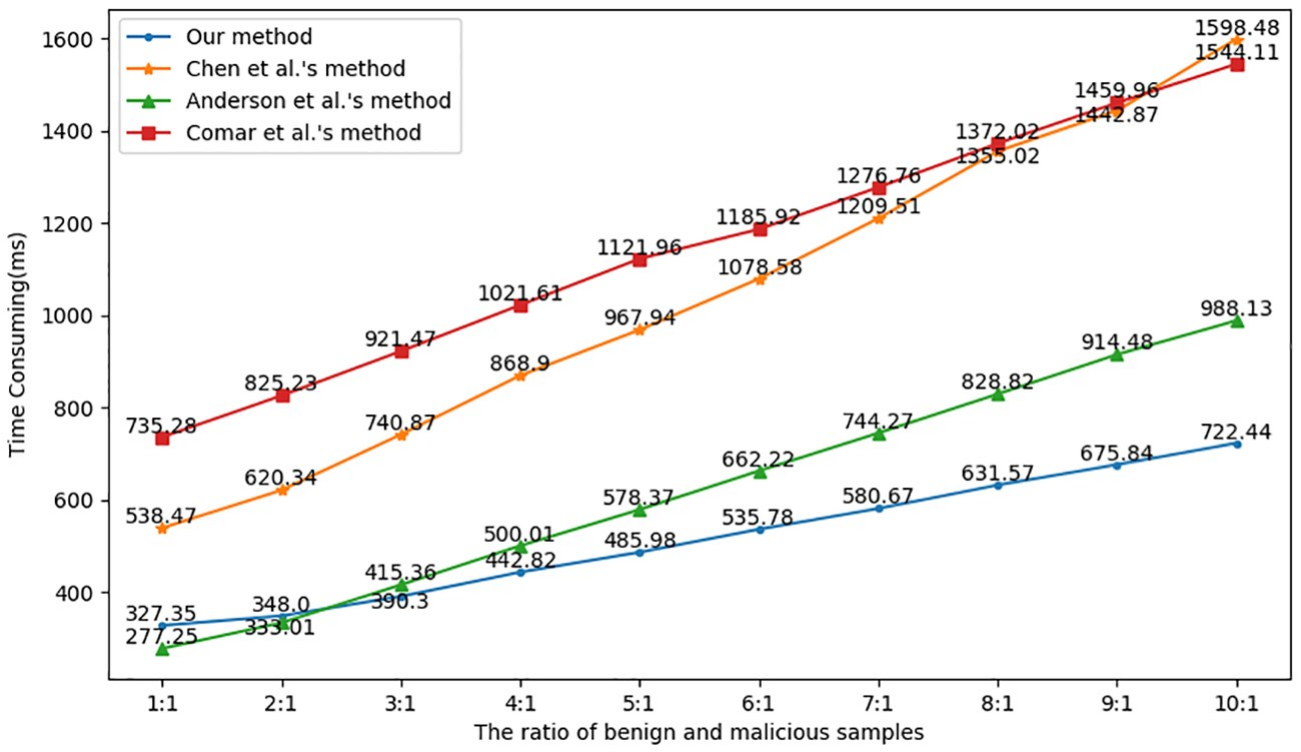
Time consumption among the 4 different methods.

We also compare our method with 3 other methods in terms of the classification efficiency. The related results are depicted in Fig 10, in which the average time consumption of each method at different sample ratios is calculated.

As seen from the figure, Anderson et al.’s method [29] utilizes a single-layer detection framework, and their method is more efficient than ours when the sample ratio is not more than 2:1. However, their method is of low efficiency when the sample ratio is over 2:1. The reason could be that the number of features they used is less than that in the second layer of our method but more than that in the first layer of our method. Comar et al.’s method [6] is based on a two-layer detection framework, in which the first layer is also used to exclude benign flows. However, the second layer consists of a set of 1-class SVM models to identify a specific malware class, which means that a potential malicious flow needs to traverse all the models before obtaining the classification result. Though the number of features is less than in our method, the time consumption is always higher than ours. Chen et al.’s method [5] proposed a triple-layer detection framework; the additional layer is the second layer, which is used to recognize the attack type. That is, a potential malicious flow needs to be classified twice, which adds extra time for detection. Thus, the efficiency of Chen et al.’s method is always less than ours.

In summary, though the efficiency of a classifier is strongly related to the number of features, our two-layer detection framework is more efficient than other methods that utilize fewer features. There are two reasons: 1) our method utilizes a multiclassifier to identify the malware family, which is more efficient than the set of classifiers used by Comar et al.’s method. 2) We used fewer features in the first layer; as long as the number of features in the filtering model is less than that of other methods (such as Anderson et al.’s method), our method is more efficient than other methods with increasing ratios of benign and malicious samples.

## Conclusion

The TLS protocol as a kind of cryptographic protocol is increasingly employed to establish the C&C channel by malware. The identification of malicious TLS flows is becoming an inevitable challenge. In this paper, we proposed a two-layer framework that exhibited high accuracy and superior efficiency. The first layer is the filtering model, which consists of a BC based on a new set of TLS handshake features and is used to filter out benign TLS flows, while the second layer is devised to identify the malware family via both TLS handshake features and statistical features. The reliability of the filtering model is demonstrated via contrast experiments, through which 96.32% of benign TLS flows are filtered out with all malicious TLS flows left. Moreover, for dealing with the multiclassification problem, we compare the effects between a multiclassifier and a set of binary classifiers under the same feature set. Experiments show that the multiclassifier performs better both in detection efficiency and in detection accuracy. Upon combining the filtering model and the malware family classification model, the high accuracy and superior efficiency of the proposed two-layer detection framework are substantiated by comparison experiments.

During our research, we also observed that the filtering model has the ability to detect unknown malicious TLS flows. Since we find substantial discrimination between benign and malicious TLS flows in the handshake phase, there is a chance to recognize unknown malicious TLS flows. In upcoming research, we plan to redesign further experiments to prove this idea.

## Supporting information

S1 File Selected binary classifier feature set data. This is the data of feature set used in filtering model.
(CSV)

S2 File Selected multiclassifier feature set data. This is the data of feature set used in malware family classification model.
(CSV)

S1 Data (PDF)
