# Supplementary material for: Two-layer detection framework with a high accuracy and efficiency for a malware family over the TLS protocol
Source: PLoS One. 2020 May 6;15(5):e0232696. doi: 10.1371/journal.pone.0232696 (PMC7202608; doi:10.1371/journal.pone.0232696)
Supplement: S1 Data — (PDF) [file pone.0232696.s003.pdf]

Two-layer Detection Framework with a High Accuracy and Efficiency for a Malware Family over the TLS Protocol

Rongfeng Zheng[1◑], Jiayong Liu[2◑*], Liang Liu[2‡], Shan Liao[2‡], Jihong Wei[2‡], Kai Li[2‡], Li Li[2‡], Zhiyi Tian[2‡]

1 College of Electronics and Information Engineering, Sichuan University, Chengdu, China.
2 College of Cybersecurity, Sichuan University, Chengdu, China.

◑These authors contributed equally to this work.
‡These authors also contributed equally to this work.
¤Current Address: Dept/Program/Center, Institution Name, City, State, Country
†Deceased
¶~~Membership~~ The membership list can be found in the Acknowledgments section.
* ljy@scu.edu.cn

Abstract

~~Transport Layer Security~~ The transport layer security (TLS) protocol is widely adopted by apps as well as malware. With the geometric growth of TLS traffic, accurate and efficient detection of malicious TLS flows is becoming an imperative. However, current ~~researches either focus on~~ studies focus on either detection accuracy or detection efficiency, and few studies take into account both indicators. In this paper, we propose a two-layer detection framework composed of a ~~Filtering Model~~ filtering model (FM) and a ~~Malware Family Classification Model~~ malware family classification model (MFCM). In the first layer, a new set of TLS handshake features is presented to train the FM, which is devised to filter out a majority of benign TLS flows. For identifying malware families, both TLS handshake features and statistical features are applied to construct the MFCM in the second layer. Comprehensive experiments are conducted to substantiate the high accuracy and efficiency of the proposed two-layer framework. A total of 96.32% of benign TLS flows can be filtered out by the FM with few malicious TLS flows being discarded provided the threshold of the FM is set to 0.01. Moreover, a ~~multi-classifier~~ multiclassifier is selected to construct the MFCM ~~in terms of better performance compared with~~ to provide better performance than a set of binary classifiers under the same feature set. In addition, when the ratio of benign and malicious TLS flows is set to 10:1, the detection efficiency of the two-layer framework is 188% faster than that of the single-layer ~~one~~ framework, while the average detection accuracy reaches ~~to~~ 99.45%.

Author summary

Rongfeng Zheng received ~~a master'~~his master's degree from Sichuan University, China, in 2016. ~~He is currently pursuing the~~ Since then, he has been pursuing a Ph.D. degree in the Electronic Information College of Sichuan University~~since 2016.~~ . His primary research interests include cybersecurity, threat intelligence, malware detection, machine learning, and ~~the~~ Internet of Things (IoT) security. ~~Now, his~~ His research focuses on

detecting malware traffic by combining ~~machine learning-related~~ machine-learning-related methods, especially on ~~those malware utilized~~ malware that utilizes open communication protocols such as DNS, HTTP, and TLS to establish ~~Command and Control~~ command and control (C&C) channels. In the past few years, he has published 3 papers: 1. “Homology analysis of malicious code based on dynamic-behavior fingerprint”, Journal of Sichuan University (Natural Science Edition), 2016.7. 2. “Requested Domain Name-based DNS Covert Channel Detection”, Netinfo Security, 2019.8. 3. “A Distance-based Method for Building an Encrypted Malware Traffic Identification Framework”, IEEE Access, 2019.7.

Introduction

~~The plaintext~~ Plaintext messages can be readily eavesdropped and tampered with during transmission, which poses a great security risk to network users. ~~The~~ This plaintext access behavior has been marked as unsafe by Google Chrome. In this context, the ~~TLS~~ transport layer security (TLS) protocol has been widely adopted for its ability to encrypt ~~the~~ plaintext and to prevent general man-in-the-middle attacks as mentioned in [?]. According to ~~the latest~~ Sandvine's latest report [?], encrypted traffic ~~has accounted~~ accounts for 50% of global web traffic.

The TLS protocol can guarantee the security of users' access to the Internet~~,~~; however, it also facilitates malware to establish command and control (C&C) channels. Malware can briskly pass through the firewall via TLS-based communication technology, and the encrypted payload makes it difficult to analyze. Malicious TLS traffic ~~is also showing~~ has also shown an increasing trend in recent years. As portrayed in Cisco's report in 2018 [?], 33% of malware ~~utilize~~ utilizes the TLS protocol to establish C&C communication. In addition, MITRE ATT&CK [?] ~~records~~ has recorded a series of cyber attacks exposed in the past few years, and the number of attacks using 443 ports to establish C&C communication accounts for 66.67%. Therefore, the wide application of the TLS protocol brings a ~~big~~ large challenge to achieve the purpose of identifying malicious TLS flows with ~~superior~~ suitable efficiency.

In ~~the industry,~~ industry, the whitelist approach has played an indispensable role in refining malware detection efficiency. Through checking ~~Server Name~~ the server name field or domain in ~~Certificate~~the certificate, the TLS flows regarded as ~~"benign"~~ “benign” can be filtered out directly. Nevertheless, ~~Server Name and Certificate~~ server names and certificates can be fabricated by malware, which makes the whitelist approach unreliable to some extent.

Facing this sophisticated and untrusted communication environment, this paper proposes a two-layer detection framework with a rapid rate and high ~~preciseness~~ precision based on the supervised learning algorithm. Current ~~researches are either focusing on the improvement of~~ studies focus on either improving the detection accuracy [?, ?, ?] ~~, or focusing on~~ or optimizing the detection efficiency [?, ?]. ~~And few~~ Few studies discuss how to improve the detection efficiency for a two-layer detection framework without affecting the detection accuracy. Indeed, as long as a majority of benign TLS flows are excluded quickly, both detection indexes can be guaranteed. ~~Meanwhile~~Moreover, through further exploration of the features of TLS flows, we can establish a more accurate classification model. Accordingly, we ~~proposed two modelsnamely Filtering Model and Malware Family Classification Model. One~~ propose two models, namely, a filtering model and a malware family classification model. The former is applied to filter out a majority of benign TLS flows, ~~the other~~ and the latter is employed to identify malware families. ~~Combining~~ The combination of these two models ~~can form~~ forms our two-layer detection framework. The innovations of this paper are ~~mainly~~ as follows:

1) A binary classifier termed ~~Filtering Model~~ the filtering model based on a new set of TLS handshake features is constructed, in which the accuracy (ACC) and the false positive rate (FPR) can reach 99.82% and 0.072%~~respectively. Meanwhile, when~~ , respectively. When the threshold of ~~classifier sets~~ the classifier is set to 0.01, the ~~Filtering Model~~ filtering model can exclude 96.32% of benign TLS flows in advance without affecting the identification of malicious TLS flows.

2) Comparison experiments are conducted between a ~~multi-classifier~~ multiclassifier and a set of binary classifiers under the same feature set to select a better method of dealing with a ~~multi-classification problem. And the~~ multiclassification problem. The superior performance of the ~~multi-classifier~~ multiclassifier is verified through comparison experiments.

3) This paper proposed a two-layer framework to refine the efficacy of detecting TLS flows, in which the first layer applies a binary classifier to filter out benign TLS flows ~~;~~ and the second layer employs a ~~multi-classifier~~ multiclassifier to identify the malware family of TLS flows. Experiments show that our two-layer framework can greatly improve the detection efficiency, while the detection accuracy is also guaranteed.

The remainder of this paper is arranged as follows. Related work is described in Section 2. ~~Problem~~ A problem statement is introduced in Section 3. Section 4 shows the two-layer detection framework. Section 5 introduces the TLS protocol, especially the TLS handshake information. Section 6 ~~is about~~ discusses feature engineering, including TLS handshake features, statistical features, and feature selection ~~method~~methods. Section 7 ~~manifests~~ presents the experiments and the related remarks. ~~Conclusion~~ The conclusion is demonstrated in the last section~~as well as the future works~~, in which potential future work is also discussed.

Related work

For encrypted network traffic, effective identification cannot be done via simply matching signatures used by traditional ~~Deep Packet Inspection~~ deep packet inspection (DPI) methods. Because the encrypted payload does not have ~~the~~ a fixed string, ~~such DPI tools like Snort [?] are not able to~~ DPI tools such as Snort [?] do not work. To remedy ~~the drawback, many efforts have~~ this drawback, much effort has been devoted to building various detection models via statistical features [?, ?, ?, ?, ?, ?], such as the packet size, ~~the~~ number of packets and ~~the inter-packed~~ interpacked time.

Some works ~~focus~~ have focused on discovering and selecting more relevant features among statistical features. A feature selection method utilizing correlation ~~is~~ was proposed by Wang et al. [?], in which the least feature set ~~is~~ was selected based on KDD Cup 99 dataset [?] and NSL-KDD dataset [?]~~and superior detection efficiency is gainedcompared with the other method. In the study of~~ , and high detection efficiency was gained. In a study by McGaughey et al. [?], the fast orthogonal algorithm ~~is applied to selecting~~ was applied to select 12 features from 2839 features, which reduced the time overhead by 81% while maintaining the detection rate~~is also guaranteed~~. Zhang et al. [?] ~~propose~~ proposed two feature selection algorithms. One is called ~~”~~ “WSU_AUC~~”~~ ” and is used to deal with the class imbalance problem; the other is termed SRSF and is employed to select robust and stable features. ~~And the~~ The advancements of the classification model ~~are testified by the~~ were verified by experiments. Optimizing the feature set can improve the detection efficiency. Nonetheless, ~~when it comes~~ in regard to the encrypted network traffic, the classification models based only on the statistical features ~~is not enough~~ are insufficient to detect malicious traffic because there exist many false positives ~~which~~ that are difficult to analyze.

In the identification of malicious encrypted traffic, some works have also explored

other detection methods. Chen et al. [?] ~~design a multi-layer detection framework which employed to ease~~ designed a multilayer detection framework that was employed to alleviate the class imbalance problem. To improve the detection accuracy, they proposed ~~the tree-Shaped Deep Neural Network~~ a tree-shaped deep neural network algorithm along with ~~the Quantity Dependent Back-propagation~~ a quantity-dependent backpropagation algorithm to establish a detection model ~~via~~ based on statistical features. Experiments ~~show that it~~ showed that this model could achieve higher detection accuracy ~~compared with~~ than other methods. Comar et al. [?] ~~design~~ designed a two-layer detection model ~~,~~ and focused on introducing a tree-based feature transformation algorithm to obtain more effective features. The main function of the first layer ~~is~~ was also to filter out benign packets, but there ~~is~~ was no detailed description of the filtering mechanism, and they did not evaluate whether the method they proposed could improve the detection efficiency. Celik et al. [?] ~~identify~~ identified malware by heartbeat packets. Zhao et al. [?] ~~detect~~ detected APT attack traffic by analyzing DNS records. Vadrevu et al. [?] ~~capture~~ captured malicious flows by identifying download behaviors produced by malware. Bilge et al. [?] ~~use~~ used Netflow [?] records in conjunction with an external evaluation system to detect malware C&C communications. However, all these ~~researches depicted above are focusing~~ studies depicted above focus on how to refine the detection accuracy and seldom ~~discussing~~ discuss the impact on detection efficiency.

Since the TLS protocol exchanges plaintext information during the handshake phase, more reliable features can be brought to construct the classification model. Cisco engineers Anderson and David et al. have conducted in-depth ~~researches~~ research on malicious TLS flows. Their main contributions are exploring various new features that can be applied to improve the detection accuracy of TLS flows [?, ?, ?]. In [?], the state transition features based on the Markov chain and the byte distribution features are verified by contrast experiments. Context information including DNS responses, HTTP headers, and TLS handshake information are imported to establish classification models of a malware family in [?]. ~~In [?] , they~~ The authors of [?] further discuss TLS handshake characteristics and combine the other 3 kinds of statistical features to detect malicious TLS flows. In the ~~researches~~ studies mentioned above, ~~they~~ the authors all claim that their methods significantly increase the performance of classifiers. However, in ~~their latest~~ the recent study [?], by ~~only using~~ using only the TLS handshake features, the accuracy of the two-class model ~~is~~ was determined to be 98.2%. When the false discovery rate is ~~at~~ 0.01%, the accuracy is 63.8%, which means that ~~their~~ this method produces many false positives. In the process of reproducing ~~their method ,~~ the method of Anderson et al., we found that only a few TLS handshake features are ~~taking~~ taken into consideration and that TLS handshake features can be further mined. Moreover, there is no detailed discussion on the detection efficiency in ~~their~~ these papers. Accordingly, ~~inspired by their~~ motivated by this prior research, we can train ~~Filtering Model by only using the~~ a filtering model by using only TLS handshake features and establish ~~Malware Family Classification Model~~ a malware family classification model by utilizing both TLS handshake features and statistical features.

In fact, some researchers are ~~dedicating to improve~~ dedicated to improving the detection efficiency of network traffic. Liya et al. [?] ~~use~~ used a hierarchical clustering algorithm to divide the samples into multiple clusters. Several representative flows are selected in each cluster. The classification result of these flows is the classification result of the entire cluster by applying ~~Multinomial Naive~~ the multinomial naive Bayes algorithm. In this way, the detection efficiency can be improved because many flows do not need to be classified. ~~But, a little loss about~~ However, a small loss in accuracy does exist in the related experiments. Wang et al. [?] ~~present the Seed-Expanding~~ presented the seed expanding (SE) algorithm to optimize clustering performance, which can

significantly reduce the number of iterations when two seeds ~~were~~ are selected. However, there ~~is no further discussing~~ was no further discussion of the influence of the detection effect. Most of the ~~works manifested previously deal with large~~ previous works deal with heavy network traffic via clustering-related methods~~, there~~. There are few discussions on improving efficiency by designing a reasonable detection framework ~~only based~~ based only on the supervised learning algorithm, and this is exactly what this paper ~~is going~~ aims to do.

Problem statement

~~For detecting~~ To detect malicious TLS flows efficiently, this paper proposes a two-layer detection framework. The first layer is designed to filter out benign network traffic; the second layer is utilized to identify malware families of TLS flows. Similar detection frameworks are used in [?] and [?], but in their methods, neither any description of the filtering mechanism nor the efficiency evaluation is mentioned. Simultaneously, the TLS flow is a kind of encrypted network traffic and cannot be filtered by simply matching the signature. For the proposed two-layer detection framework, in addition to the extra ~~consuming~~ consumption time of the ~~Filtering Model~~filtering model, the traversal times of the two-layer framework are also more than that of the single-layer ~~one~~framework, which may result in ~~that~~ the two-layer framework ~~is~~ being less efficient than the single-layer framework. To ~~cover~~ address this disadvantage, the ~~consuming~~ consumption time of the ~~Filtering Model~~ filtering model must be lower than that of the ~~Malware Family Classification Model~~malware family classification model. Accordingly, the first problem is how to train an efficient ~~Filtering Model~~ filtering model (a binary classification model~~) which~~ , BC) that can filter out benign TLS flows with a rapid rate and high ~~preciseness properties~~precision.

Due to the existence of various malicious TLS flows in cyberspace, the ~~Malware Family Classification Model mainly focuses~~ malware family classification model focuses mainly on solving a ~~multi-classification~~ multiclassification problem. To accurately identify the malware family of TLS flows, either a ~~multi-classifier or the ”~~multiclassifier or the “one against all~~”~~” strategy that utilizes a set of binary classifiers can be applied. However, ~~the~~ current studies seldom compare the effects of these two options under the same feature set in the field of network flow detection. Hence, the second problem is which option is better to deal with the ~~multi-classification~~ multiclassification problem.

Two-layer detection framework

In a real ~~Gigabit~~ gigabit network environment, hundreds of TLS flows generated ~~in~~ every minute make it costly to identify malware families of TLS flows in real time. ~~Besides~~In addition, as the number of malware families surges, so does the pressure ~~of~~ on the detection system. Hence, it is imperative and ~~worthy to design~~ appropriate to design a detection framework to reduce the time consumption of TLS flows and guarantee the detection accuracy at the same time.

We propose a two-layer detection framework as shown in Fig~~1, the~~. 1. The first layer consists of a ~~binary classification model termed Filtering Model~~BC termed the filtering model, which is ~~mainly applied~~ applied mainly to filter benign TLS flows based only on TLS handshake features~~; the~~. The second layer is a ~~Malware Family Classification Model~~ malware family classification model for identifying the malware family of TLS flows based on both TLS handshake features and statistical features. When a new TLS flow is imported into this detection framework, the detection process is as follows.

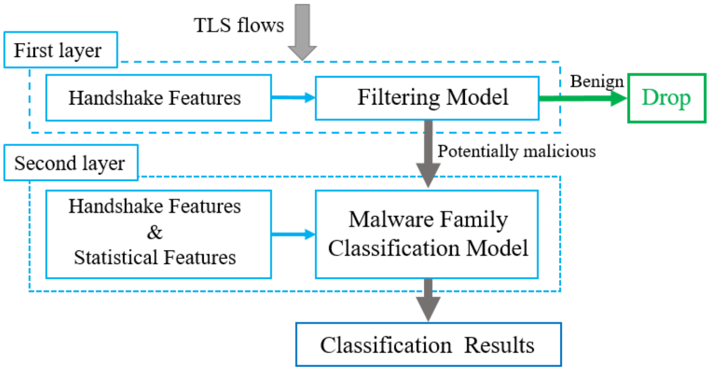

Fig 1. Two-layer ~~Detection~~ detection framework.

~~It~~ The flow is sent to the ~~Filtering Model; if the Filtering Model discriminates~~ filtering model; if this model identifies it as a benign TLS flow, it is directly discarded and no longer put into the next layer; if ~~being~~ classified as a potentially malicious TLS flow, it passes to the next layer for further identification about which malware family it belongs to. Through this process, one can speculate that the TLS flow ~~which~~ that is not discarded by the ~~Filtering Model~~ filtering model may contain both malicious TLS flows and benign TLS flows. ~~But~~ However, compared to the number of flows in the first layer, the number of benign TLS flows in the second layer ~~will be much lessthan that in the first layer, thus~~is much less; thus, the detection efficiency can be improved.

~~For making~~ To make the two-layer framework more efficient, ~~it requires that the consuming time of~~ the time consumed by the first layer ~~is~~ must be less than that of the second layer~~,~~; otherwise, the two-layer framework would reach the opposite destination. In this section, an ~~inequation~~ inequality is used to infer the condition with superior efficiency by the mathematical calculation concerning the ~~consuming time of~~ time consumed by the two models~~, respectively. If our method is more efficient , which means the time overheadof our method is less. Considering the following inequation~~. A more efficient method will result in lower time overhead. We consider the following inequality:

$$NF * T_1 + (1 - r) * NF * T_2 < NF * T_2 \quad (1)$$

In Ineq. (1), NF represents the number of TLS flows, T_1 represents the average ~~consuming time of Filtering Model~~ time consumed by the filtering model for every piece of flow, r represents the proportion of TLS flows ~~which is~~ filtered out by the first layer ($r \in [0, 1]$), and T_2 represents the average ~~consuming time of Malware Family Classification Model for every piece of flow . This inequation~~ time consumed by the malware family classification model for every flow segment. This inequality can be simplified as follows:

$$r > T_1 \setminus T_2 \quad (2)$$

From the ~~inequation~~inequality, we can ~~get the conclusion~~ conclude that the efficiency of our method does ~~not depend~~ depends not on the number of flows ~~,~~ but on the proportion of flows filtered out by the first layer. The original range of r is $[0, 1]$. To make the two-layer framework more efficient, the value of T_2 must be greater than T_1. Under this condition, the range of r needs to ~~belong to~~ be $(T_1 \setminus T_2, 1]$.

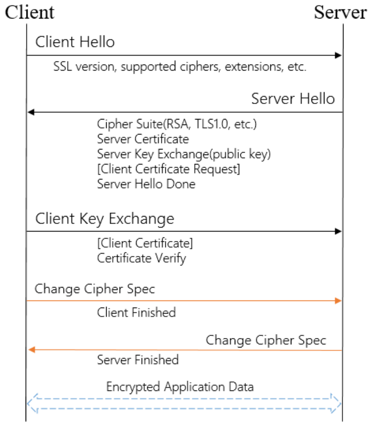

Fig 2. TLS protocol key negotiation process.

Since the model of the first layer has fewer training features ~~, the Filtering Model consuming time~~ than the malware family classification model, the time consumption T_1 of the former is less than the ~~Malware Family Classification Model consuming time~~ time consumption T_2 ~~. That is to say~~of the latter. That is, if the prerequisite condition of Ineq. (2) is satisfied, a more efficient detection process can be achieved.

TLS ~~Handshake Information~~handshake information

The TLS protocol is ~~called Transport Layer Security which is~~ derived from the ~~Secure Sockets Layer~~ secure sockets layer (SSL) protocol. ~~Now, the~~ The TLS protocol version has been updated to 1.3, but the mainstream version is still 1.2. Few apps implement ~~the 1.3 version~~ version 1.3, which regulates the samples collected in this paper ~~mainly based~~ to be based mainly on TLS 1.0, TLS 1.1 and TLS 1.2.

Fig. 2 shows a typical process for TLS key negotiation. In this process, two main purposes are completed, namely, key negotiation and identity authentication, and the message information exchanged between the client and the server is the focus in this paper. In ~~Fig 2, the Client Hello contains~~ the figure, the client hello contains the TLS version, ~~Cipher Suites, Extensions~~cipher suites, extensions, etc. The ~~Server Hello includes~~ server hello includes the TLS version, ~~Cipher Suite, Extensions, Certificate, Server Key Exchange, and Client Certificate Request~~cipher suite, extensions, certificate, server key exchange, and client certificate request. In the ~~Change Cipher Spec,~~ change cipher specification, since the message between the client and server is very ~~fixed~~constrained, this paper does not consider extracting features from it. In fact, ~~lots of~~ much plaintext information is exchanged in the key negotiation phase except for a few encryption fields.

~~Although~~ Because the negotiation information generated by different ~~softwares~~ software programs is not completely the same, such as ~~Cipher Suites, Server Name, and Certificate~~ the cipher suites, server name, and certificate information, it is not feasible to extract the signature features that can be used to identify the TLS flows. ~~Because different~~ Different applications may also adopt the same ~~Cipher Suite~~ cipher suite and other negotiation information.

~~For saving~~ To save computing resources of the server, malware ~~is generally more inclined~~ tends to adopt simple encryption algorithms and provides ~~less~~ little handshake information [?], which allows benign applications and malware to show many differences

during the key negotiation phase.

Feature engineering

TLS handshake feature

In Anderson et al.'s method [?], three main types of features are used: the list of offered ~~Cipher Suites~~cipher suites, the list of advertised extensions, and the public key length. A total of 198 TLS handshake features are selected in their method. ~~But~~However, in the TLS key negotiation phase, there ~~not only show differences~~ are differences not only in these fields but also in other fields~~. Such as Protocol Version, Server Name, Client Hello Length, Cipher Suites Number, Extension Number, and Certificate Number~~, such as the protocol version, server name, client hello length (CHL), cipher suite number, extension number, and certificate number. This paper compares the ~~discriminations~~ discrimination between benign and malicious samples ~~on~~ in these fields (~~Refer to Data~~ refer to the discussion of data collection for details about the sample set).

Protocol ~~Version: the~~ version: The protocol version used by most of the benign applications is TLS 1.2, and the TLS flows with lower protocol version ~~only accounts for~~ account for only 2.19% of the entire TLS flows. ~~But~~ However, among malicious TLS flows, the proportion of the lower protocol version is higher, reaching 30.28%.

Server ~~Name: there~~ name: There are different forms in the representation of this field. This field may be empty, ~~may be filled with GDA (Domain Generation Algorithms)~~ filled with the domain generation algorithm (GDA) domain, or ~~may be~~ filled with IP ~~address. The~~ addresses. The corresponding proportions are 0.51%, 17.77%, and 1.32% in the benign TLS flows~~, respectively; but~~ ; however, in malicious TLS flows, ~~the~~ these proportions are 71.36%, 4.0%, and 0%.

Other fields: since these fields are all represented by numerical values, we group them ~~to describe for convenience~~ for convenience of description. In general, malware ~~have smaller values on~~ has smaller values in these fields, while benign TLS flows tend to have ~~longer Client Hello Length, larger Cipher Suites Number, Extension Number, and Certificate Number~~a longer CHL and a larger cipher suite number, extension number, and certificate number. From these fields, some features are selected to draw Fig. 3 according to 2 ~~criterias~~criteria: 1) the value of each feature is ~~large~~ larger than 0.05~~;~~ , and the ratio of benign ~~and~~ to malicious (or malicious ~~and~~ to benign) samples at each feature is ~~large~~ larger than 3. The features ~~which~~ that satisfy both criteria can be selected ~~into Fig~~ in Fig. 3. ~~As shown in Fig3, a trend can be seen~~ A trend is observed that as the numerical value is larger, the proportion of malicious TLS flows is lower, while the proportion of benign TLS flows is higher.

In addition, sparse representation is applied to design the features of each field. For example, under the ~~Client Hello Version~~ client hello version field, we set 3 features, namely, TLS 1.0, TLS 1.1 and TLS 1.2. Under the ~~Client Hello Length (CHL)~~ CHL field, we set 150 bins of 10 bytes each, from [0, 10) to [1490, 1500)~~,~~ ; if the CHL is greater than 1490, it ~~will be~~ is put into the last bin. Each bin represents a feature, so there are a total of 150 features in the CHL field. The value of CHL belongs to ~~which bin,~~ a certain bin; this bin's value ~~will be~~ is 1, and the ~~rest will be~~ remaining values are 0. These features are called ~~'sparse feature'~~ "sparse features", and we use ~~it~~ them to devise our feature set. In addition to the features proposed by Anderson et al. [?], we further ~~mined~~ mine the other 6 kinds of TLS features~~including Client Hello Version, Client Hello Length, Cipher Suites Number, Client~~, including the client hello version, CHL, cipher suite number, client/~~Server Extension Number, Server Name, and Certificate Number~~server extension number, server name, and certificate number. As

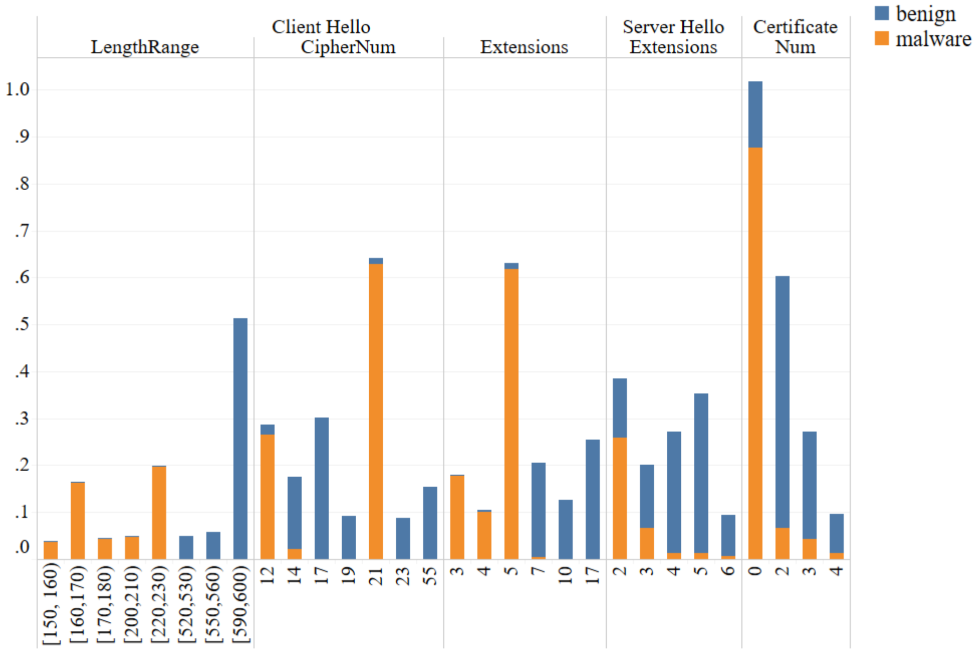

Fig 3. ~~Difference~~ Differences in other fields.

shown in Table 1, there are a total of 705 features.

Table 1. TLS handshake feature set.

**Feature Name**	**Description**
Client ~~Hello Version~~ hello version (*new*)	Which version it belongs to
~~Client Hello Length~~ CHL (*new*)	Which bin it belongs to (10 bytes ~~a~~ per bin)
Cipher ~~Suites Number~~ suite number (*new*)	Which number it belongs to
Client ~~Cipher Suites~~cipher suites	Which ~~Cipher Suites~~ cipher suites it belongs to
Client ~~Extension Type~~extension type	Which ~~Extension Type~~ extension type it belongs to
Client ~~Extension Number~~ extension number (*new*)	How many ~~Extensions~~ extensions it has
Server ~~Name~~ name (*partly new*)	If it is in the top 1 million DNS Alexa (*not new*), empty, rando[illegible]
~~Client Public Key Length~~Client public key length	Which ~~Key Length~~ key length it belongs to
Client ~~Signature Algorithm Number~~signature algorithm number	Which number it belongs to
Client ~~Padding Length~~padding length	Which bin it belongs to (8 bytes ~~a~~ per bin)
Server ~~Hello Version~~hello version	Which version it belongs to
Server ~~Cipher Suite~~cipher suite	Which ~~Cipher Suite~~ cipher suite it belongs to
Server ~~Extensions Type~~extensions type	Which ~~Extensions Type~~ extensions type it belongs to
Server ~~Extensions Number~~ extensions number (*new*)	How many ~~Extensions~~ extensions it has
Certificate ~~Number~~ number (*new*)	How many ~~Certificates~~ certificates it has

Statistical features

To accurately identify malware families of TLS flows, it is ~~not enough to just use~~ insufficient to use just the TLS handshake features. Anderson et al. [?] ~~have proved~~ proved that TLS handshake features combined with statistical features can achieve higher detection accuracy than other techniques in identifying malware families. Here,

we refer to the research of predecessors and select a set of statistical features that have been verified. Aksoy et al. [?] ~~utilize~~ utilized the features in packet headers to train classifiers. The validity of the packet length distribution and time interval distribution ~~are~~ is demonstrated in [?]. The first packet length and minimum packet length feature are used in [?]. The Markov chain generated by the sequence of the length and time interval among packets ~~were~~ is mentioned in [?], and the state transition probability is used as the feature. By taking advantage of the research results of predecessors, as shown in Table 2, we summarize the statistical features in this paper.

Table 2. Statistical features.

Description	Feature number
Min. packet length	2
Max. packet length	2
First packet length	2
Packets with a push flag	2
Packet ~~Length Distribution~~length distribution	150
Packet ~~Inter-arrival Time Distribution~~interarrival time distribution	100
Byte ~~Distribution~~distribution	256
Packet ~~Inter-arrival Time Transition Probability Matrix~~interarrival time transition probability matrix	100
Packet ~~Length Transition Probability Matrix~~length transition probability matrix	100

In Table 2, since we take the direction of the flow into account (client to server and server to client), ~~Min~~ the min. packet length is represented by two features, ~~and the same with Max~~ the same as the max. packet length, ~~First~~ first packet length, and ~~Packets with~~ packets with a push flag. For ~~Packet Length Distribution~~the packet length distribution, we also set 150 bins of 10 bytes each and calculate the length distribution of the first 100 packets among the 150 bins. For ~~Packet Inter-arrival Time Distribution~~the packet interarrival time distribution, we set 100 bins of 5 ~~milliseconds~~ ms each, and any ~~inter-arrival time~~ interarrival time of more than 495 ~~milliseconds will put into~~ ms is put in the last bin. Then, we calculate the ~~inter-arrival~~ interarrival time distribution of the first 100 packets among the 100 bins. For the ~~Byte Distribution~~byte distribution, we compute the ratio of each byte ~~counts~~ count to the total number of bytes in the packet payload. There are 256 representations of a byte, so there are 256 features. For ~~Packet Inter-arrival Time Transition Probability Matrix~~the packet interarrival time transition probability matrix, we set 10 bins of 50 ~~milliseconds~~ ms each, and any ~~inter-arrival time~~ interarrival time of more than 450 ~~milliseconds will put into~~ ms is put in the last bin. We calculate the transition probability matrix with the first 100 packets based on the Markov chain. Similarly, for ~~Packet Length Transition Probability Matrix~~the packet length transition probability matrix, we set 10 bins of 150 bytes each ~~, and also~~ and calculate the length transition probability matrix by utilizing the first 100 packets. The statistical features ~~will be~~ are combined with the handshake features ~~together~~ to establish a more accurate classification model for identifying malware families.

Feature selection

Because we use sparse representation to design our feature set, the produced features are high dimensional. Inevitably, there are some irrelevant features in the feature set. For this reason, before training the model, we need to reduce the number of feature dimensions by removing those irrelevant features. Because the ~~Filtering Method~~ filtering method does not depend on a specific machine learning method, it has the characteristics of high operational efficiency and is suitable for solving the problem of

feature selection in high-dimensional data. We use the information gain [?], which is one of the ~~Filtering Methods~~ filtering methods, to select more relevant features. The information gain can be expressed as the difference between the entropy and conditional entropy, as shown in the following equation:

$$IG(X) = H(C) - H(C \mid X) \tag{3}$$

In Eq. (3), H(C) stands for the information entropy, and its essence is the measure of the uncertainty of random variables. Its definition is as follows:

$$H(C) = -\sum_{i=1}^{n} P(C_i) \log_2 \, P(C_i) \tag{4}$$

In Eq. (3), H($C|X$) stands for the conditional entropy, which is a measure of the uncertainty of random variable c with a certain value of x. Its definition can be seen in Eq. (5):

$$H(C \mid X) = \sum_{x \in X} p(x) \; H(C \mid X = x) \tag{5}$$

From the above three formulas, the information gain of each feature can be conveniently computed. By comparing the information gain of each feature, the importance of features can be measured, and by filtering out the features with low information gain, the feature dimension can be reduced.

Experiments and results

To demonstrate the effectiveness of our methods, comprehensive experiments are conducted. There are mainly 4 parts: 1) ~~detailed~~ Detailed methods of collecting samples are presented in ~~Data collection~~ the data collection part. 2) ~~the Filtering Model~~ The filtering model is established and evaluated through the selection of relevant features and a reasonable threshold. 3) ~~a multi-classifier~~ A multiclassifier and a set of binary classifiers are compared to select a better method for dealing with the ~~multi-classification~~ multiclassification problem in the ~~Evaluation Malware Family Classification Model~~evaluation malware family classification model. 4) The two-layer detection framework is evaluated by comparing it with the single-layer framework.

Data collection

In this section, the ~~collecting~~ collection methods of the sample set and the necessary ~~pre-processing~~ preprocessing steps are presented ~~detailedly~~in detail. The Streamdump tool [1] we developed is used to collect TLS flows according to the quad information {srcIP, srcPort, dstIP, dstPort}. There are two ways for StreamDump to reassemble TLS packets. One is monitoring network traffic on ~~network adapter where~~ a network adapter, where the transport layer protocol is TCP and the destination port is 443. Another is directly reading .~~pacp~~ pcap files that are saved by others. During data collection, both ~~ways~~ methods are used to collect TLS flows. For collecting benign TLS flows, StreamDump is used to reassemble real-time TLS packets~~;~~, but for malicious samples ~~which~~ that are shared by others in the form of ~~pcap files.~~ .pcap files, StreamDump is utilized to extract malicious TLS flows from these files. ~~Meanwhile, the Handshake Type~~ Moreover, the handshake type field is applied to determine whether a

[1] https://github.com/NewBee119/StreamDump

TLS flow contains a complete handshake process, and TLS flow samples that do not contain the complete handshake process ~~will be~~ are discarded.

For the collection of benign TLS flow samples, we spent 15 days collecting a total of 1323667 TLS flows from our ~~Lab~~ laboratory network. Before using these samples, we need to conduct several ~~pre-processing~~ preprocessing steps on these samples. ~~Firstly~~First, there are many TLS ~~flow~~ flows without the entire TLS handshake process because of some optimization schemes, such as session tickets. However, when the connection to the server occurs for the first time or when the session ticket time runs out, the entire TLS handshake process ~~will be~~ is required to connect to the server. ~~So~~Therefore, we need to exclude the flows that do not contain the entire TLS handshake information, and 590093 flows ~~are left~~remain. In addition, ~~åß~~ to objectively reflect the differences between benign and malicious ~~TLS~~TLSs, we delete the TLS flows ~~which~~ that have the same ~~Server Name and Client Hello Length~~ server name and CHL from these samples and ~~get~~ obtain 21743 flows after this step. In fact, at this point, we still ~~can not~~ cannot guarantee that the TLS flows obtained in the previous steps are all benign, and further preprocessing is needed. This paper uses the open-source threat community AlienVault to check ~~if~~ whether the destination IP of a TLS flow is potentially malicious. We developed the check_ip tool [2] by using AlienVault's API to discover and filter out the potential malicious TLS flows, which can ensure the purity of benign samples. Through preprocessing, we selected 18241 benign TLS flows from 1323667 TLS flows, which not only improves the quality of the sample set but also alleviates the problem of class imbalance to some extent.

For the collection of malicious TLS flow samples, we collect malware traffic samples shared on the Internet by using our own crawler tool [3]. In Malware Traffic [?], we obtain 15077 TLS flow samples; in the BCIC dataset [?], a total of 210,484 TLS flows are extracted. These flows are generated in the virtual machine by executing malware. However, there is a problem that we ~~can not~~ cannot tell whether the TLS flows are generated by malware or by other, benign applications in the virtual machine. To improve the reliability of the training data, we still use AlienVault's API to filter out TLS flows ~~which~~ that are identified as benign. After these steps, we finally ~~get~~ obtain 17923 malicious TLS flows ~~which~~ that have a complete handshake phase. Accordingly, we select ~~a part of~~ some malware families to verify our method, and ~~those~~ malware families with less than 100 flows are not ~~select. As shown in Table 3, the~~ selected. The number of TLS flows for each malware family ~~can be seen.~~ is shown in Table 3.

Table 3. TLS flows in each malware family.

Malware family	Number of flows	Unique server IPs
EITest	135	53
Emotet	1898	144
Hancitor	2613	80
Nuclear	262	19
Rig	245	49
Trickbot	1600	115
Dridex	5074	12
Razy	1019	1
HTBot	695	19

[2] https://github.com/NewBee119/check_ip
[3] https://github.com/NewBee119/malware_traffic_crawler

Evaluation of ~~Filtering Model~~the filtering model

The ~~Filtering Model~~ filtering model as a coarse classification model is ~~mainly employed~~ employed mainly to quickly filter out the benign TLS flows and to ensure that the malicious TLS flows are passed to the next layer as much as possible. ~~3~~ Three steps are presented to reach this goal: 1) selecting the relevant TLS handshake features; 2) verifying the effectiveness of the ~~Filtering Model;~~ filtering model; and 3) selecting the appropriate threshold for the ~~Filtering Model~~filtering model.

Since we ~~get~~ obtain 705 TLS ~~Handshake~~ handshake features, the feature dimension needs to be reduced before the training model. Based on the information gain algorithm mentioned in the previous section, we can calculate the information gain value (IGV) for each feature ~~,~~ and select candidate feature sets based on the IGV. The detailed process is presented in Algorithm 1. ~~Modified Wrapper~~ The modified wrapper method with a backward selection strategy is used to select the best feature subset. The information gain of each feature should be calculated in advance. IG(F_i) represents the result of information gain for feature subset F_i, F_i represents the ith feature subset, and F_0 represents the original feature set. ~~Accuracy (ACC) and false positive rate (FPR)~~ The ACC and FPR can be calculated by the classifier. $X_{labeled}$ represents the labeled benign samples and malicious samples.

Algorithm 1 Modified ~~Wrapper Method For Feature Selection~~wrapper method for feature selection.

Require: F_0, $IG(F_0)$, $X_{labeled}$
Ensure: the ~~Best~~ best feature subset (BFS)

1: ~~Select~~ select the classifier based on the logistic regression algorithm;
2: ~~Based~~ based on IG(F_0), sort(F_0) in ~~descend orderand obtain~~ descending order, and obtain the sorted F_0';
3: ~~Calculate~~ calculate ACC_{F_0} ~~,~~ and FPR_{F_0} for $X_{labeled}$;
4: **for** backward ~~selection~~ select F_0', and obtain F_i do **do**
5:   **if** min($IG(F_i)$) is equal to 0 **then**
6:     continue;
7:   **end if**
8:   calculate ACC_{F_0}, FPR_{F_0}, and TPR_{F_0} for $X_{labeled}$;
9:   **if** $ACC_{F_i} < ACC_{F_{i-1}}$ and $FPR_{F_i} > FPR_{F_{i-1}}$ **then**
10:     $ACC_{F_i} = ACC_{F_{i-1}}$, $FPR_{F_i} = FPR_{F_{i-1}}$;
11:     **if** BFS is $NULL$ **then**
12:       $BFS = F_{i-1}$;
13:     **end if**
14:   **else** $\{ACC_{F_i} \geq ACC_{F_{i-1}}$ or $FPR_{F_i} \leq FPR_{F_{i-1}}\}$
15:     $BFS = F_i$;
16:   **end if**
17: **end for**
18: **return** BFS

There are ~~mainly three~~ three main steps in Algorithm 1: 1) preparatory work (1-2); 2) calculating the initial parameters based on classifier (3); 3) evaluating F_i and selecting the best ~~features~~ feature subset (4-17). In step 3, the backward selection strategy is used to construct a feature subset (F_i), and the number of features in F_i is 1 less than that in F_{i-1}. The features in which ~~their~~ the IGV is 0 can be directly excluded because they have no contribution to the classifier (5-7). Different from the original ~~Wrapper Method,~~ wrapper method, our proposed Algorithm 1 ~~we proposed~~ can skip irrelevant features and screen out feature ~~subset~~ subsets with the highest detection accuracy. The feature subset ~~which~~ that can achieve the highest ACC can be

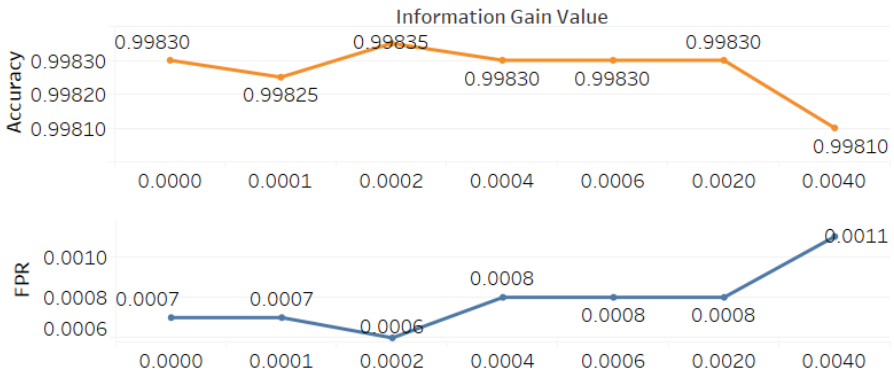

Fig 4. Classification results among different feature sets.

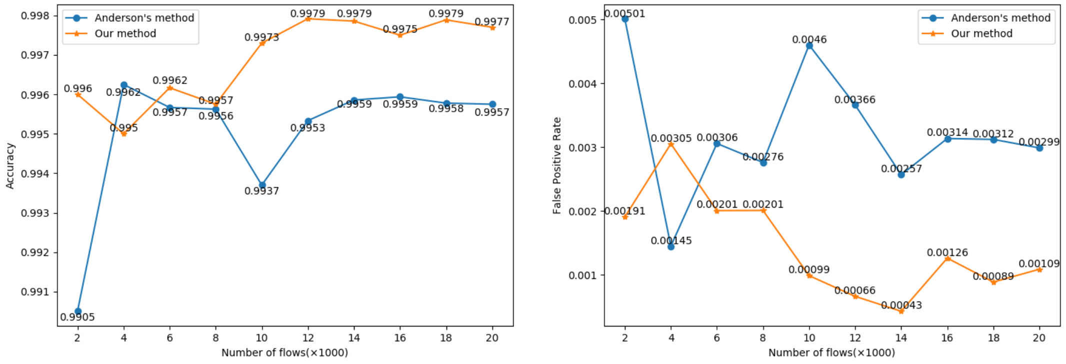

Fig 5. Comparison of the two methods.

regarded as the best feature subset (8-16). To alleviate the class imbalance problem [**?**], we randomly select 10,000 benign samples and 10,000 malicious samples and utilize the logistic regression algorithm to evaluate the performance among different feature subsets by calculating the ACC and FPR. As shown in Fig. 4, we select 7 feature subsets to ~~exhibits~~ exhibit the process described in Algorithm 1, from the feature subset in which the minimum IGV is equal to or greater than 0 to the feature subset in which the minimum IGV is equal to or greater than 0.004.

Fig. 4 shows the feature subset in which the minimum IGV is equal to or greater than 0.0002, from which we can ~~get~~ obtain the best classification results in which the ~~accuracy~~ ACC is the highest and the FPR is the lowest compared with other feature subsets. Under this condition, 297 effective features can be screened out and used to train our ~~Filtering Model~~filtering model.

For comparison, we completely reproduce Anderson et al.'s method [**?**] by utilizing the logistic regression algorithm to train classifiers and 10-fold cross-validation to evaluate the performance. The features used in our method ~~are including~~ include the 6 kinds of features we proposed; the features without a *new* tag in Table 1 are used by Anderson et al.'s method. The ~~accuracy and false positive rate~~ ACC and FPR are calculated by adopting their method and our method among different numbers of TLS flow samples~~, respectively~~.

Fig. 5 shows the comparison results of the two methods with the sample ~~ranged~~ number ranging from 2,000 to 20,000. The ratio of positive and negative samples is 1:1. When the sample size ~~is greater than~~ increases past 10,000, both the ~~accuracy and false~~

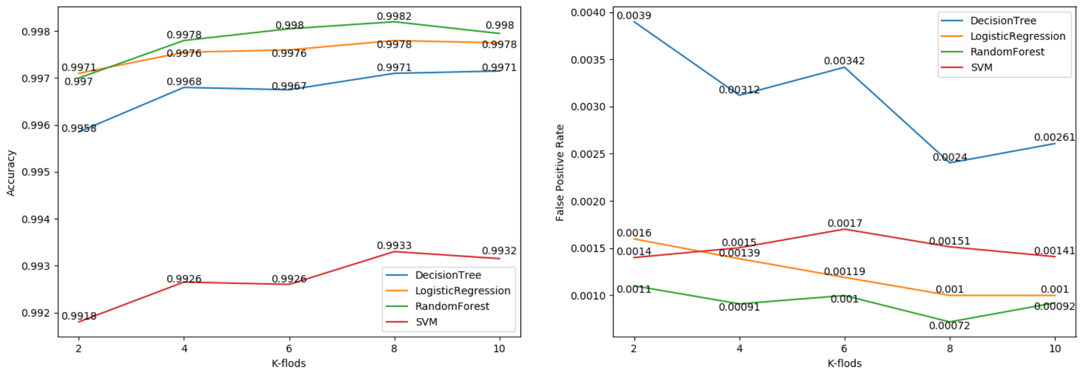

Fig 6. Comparison among the 4 different algorithms.

~~positive rate are~~ ACC and FPR become gradually stable, and we can calculate the average ~~accuracy and average false positive rate~~ ACC and average FPR under this condition. By applying the 6 kinds of features we newly proposed, the average ~~accuracy~~ ACC of our classifier is 99.78%, and the average ~~false positive rate~~ FPR is 0.09%. Compared with Anderson et al.'s method [**?**], the average ~~accuracy~~ ACC of our method is 0.20% higher than that of their method, while the average ~~false positive rate~~ FPR is 0.22% lower than their method. Since the average ~~accuracy of Anderson~~ ACC of Anderson et al.'s method is very high~~which reaches~~ , reaching 99.58%, the 0.2% improvement is also considerable. Therefore, by further mining TLS handshake features, we can establish a better ~~binary classification model compared with Anderson~~ BC than Anderson et al.'s method [**?**].

Moreover, we also compare the classification effects among different machine learning algorithms. A total of 20,000 samples with the same number of benign and malicious TLS flows are used to calculate the ~~accuracy and false positive rate~~ ACC and FPR under k-fold cross-validation. As shown in Fig. 6, all 4 algorithms can achieve ~~high accuracy~~a high ACC, but the performance of the random forest algorithm is the best both in ~~accuracy and in false positive rate with the accuracy~~ the ACC and in the FPR, with the ACC being 99.82% and the ~~false positive rate~~ FPR being 0.072%. ~~So~~ Therefore, we select the random forest algorithm to train our ~~Filtering Model~~filtering model.

The contribution of features ~~also can~~ can also be evaluated by the classifier based on the random forest algorithm. The ~~most important 20~~ 20 most important features are shown in Table 4. The cipher suites occupy nearly ~~a half, it~~ half, which means that the ~~Client Cipher Suites~~ client cipher suites used by benign applications and malware are remarkably different since malware ~~are more inclined~~ is tends to utilize simpler algorithms to encrypt network traffic. ~~It also can be seen that there~~ There are 7 features we newly ~~proposed with~~ propose with *a new* tag in this paper, which demonstrates the effectiveness of the features we proposed.

The main function of the ~~Filtering Model~~ filtering model is to filter out benign traffic~~while all the~~ , while all malicious TLS flows need to be left. We can reach this goal by setting a reasonable decision threshold in the ~~Filtering Model~~ filtering model and use all the testing samples, including 18241 benign samples and 17923 malicious samples, to evaluate our classifier. We used 10-fold cross-validation and the random forest algorithm to calculate the confusion matrix for each threshold.

In Table 5, when the threshold is set to 0.01, the value of FN is 0, which means that all malicious TLS flows can be identified as malicious. On the other ~~side~~hand, the value of TN is 17812, which means that 17812 TLS flows ~~will not be~~ are not passed to the

Table 4. The 20 most important ~~20~~ features in the ~~Filtering Model~~filtering model.

Feature description	Importance
Client ~~Cipher Suites~~cipher suites: TLS_RSA_WITH_RC4_128_MD5	0.0920
Client ~~Cipher Suites~~cipher suites: TLS_DHE_DSS_WITH_3DES_EDE_CBC_SHA	0.0716
Client ~~Extension Type~~extension type: extended master secret	0.0557
Client ~~Cipher Suites~~cipher suites: TLS_ECDHE_RSA_WITH_AES_256_GCM_SHA384	0.0539
Client ~~Signature Number~~signature number: 2 (*new*)	0.0435
Client ~~Cipher Suites~~cipher suites: TLS_ECDHE_RSA_WITH_CHACHA20_POLY1305_SHA256	0.0390
Client ~~Cipher Suites~~cipher suites: TLS_DHE_DSS_WITH_AES_256_CBC_SHA	0.0354
Client ~~Cipher Suites~~cipher suites: TLS_RSA_WITH_AES_256_GCM_SHA384	0.0318
Client ~~Extension Type~~extension type: application layer protocol negotiation	0.0296
Client ~~Cipher Suites~~cipher suites: TLS_ECDHE_RSA_WITH_AES_128_GCM_SHA256	0.0242
Server ~~Name~~ name is not in the top 1 million DNS Alexa results	0.0234
Client ~~Cipher Suites~~cipher suites: TLS_ECDHE_ECDSA_WITH_CHACHA20_POLY1305_SHA256	0.0232
Server ~~Name~~ name is a random string (*new*)	0.0225
Client ~~Extension Type: Session Ticket~~extension type: session ticket	0.0195
Server ~~Extension Number~~extension number: 1 (*new*)	0.0172
Client ~~Cipher Suites~~cipher suites: TLS_RSA_WITH_RC4_128_SHA	0.0164
~~Client Hello Length~~CHL: [150, 160) (*new*)	0.0163
~~Client Hello Length~~CHL: [610, 620) (*new*)	0.0156
Server ~~Name~~ name is empty (*new*)	0.0150
Client ~~Extension Number~~extension number: 5 (*new*)	0.0142

second layer because they are regarded as benign, and these TLS flows account for 97.65% of the total benign TLS flows. Thus, by adopting the random forest algorithm and setting the threshold to 0.01, we can establish our ~~Filtering Model~~ filtering model based only on TLS handshake features.

Table 5. Confusion matrix among different thresholds.

Threshold	Confusion matrix			
	TP	FN	TN	FP
0.4	17909	14	18235	6
0.1	17914	9	18172	69
0.05	17928	5	18101	140
0.01	17923	0	17812	429

Evaluation of ~~Malware Family Classification Model~~the malware family classification model

Generally~~speaking~~, identifying malware families of TLS flows is a ~~multi-classification~~ multiclassification problem. To deal with ~~the multi-classification~~ this problem, there are two options to select. The first option is to train a ~~multi-classification~~ multiclassification model (MC); the second is using the ~~"~~one against all ~~"~~ strategy by training a set of ~~binary classification models (BCs)~~BCs, and each model corresponds to a kind of malware family. Experiments are designed to explore which of the two options performs better.

We prepare 9 kinds of malware families and 18241 benign TLS flows, as shown in Table 3. For the first option, we only need to train a ~~multi-classifier~~multiclassifier; for the second option, we train 10 binary classifiers in advance (9 for malware families, 1 for benign samples) ~~,~~ and select the highest probability among 10 binary classifiers as the

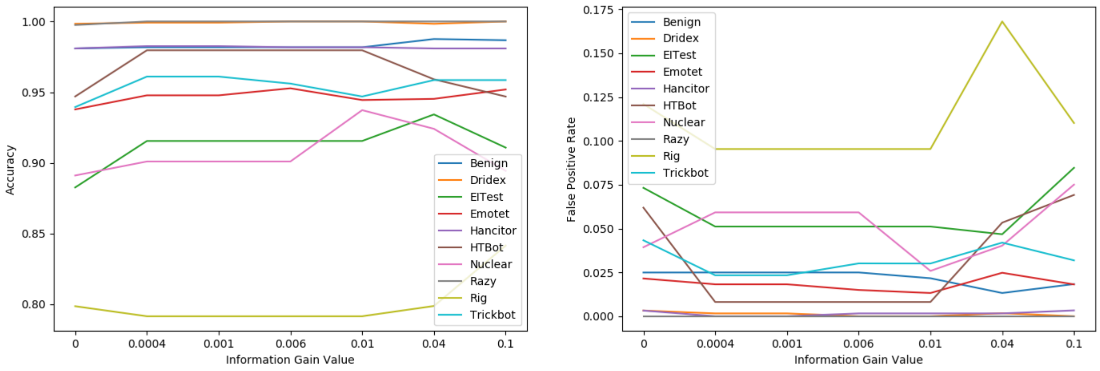

Fig 7. Classification results from 10 binary classifiers among different feature ~~set~~sets.

classification result during the test.

Before training the models, it is necessary ~~for one~~ to select relevant features from the original 705 TLS handshake features and 664 statistical features. Nevertheless, the information gain algorithm ~~can not~~ cannot be directly used to select features for ~~multi-class~~ a multiclass sample set. The feature selection method we used here contains two steps: 1) selecting relevant features for each binary classifier by utilizing the information gain algorithm ~~;~~ and 2) utilizing the union of 10 feature sets selected from 10 binary classifiers as our feature set. The process of feature selection for each binary classifier is the same as that in the ~~Filtering Model~~filtering model. As shown in Fig. 7, the ~~accuracy and false positive rate~~ ACC and FPR of each binary classifier are calculated among different feature sets.

After completing these two steps~~mentioned above~~, we finally obtain 762 features, including 234 TLS handshake features and 528 statistical features, and use the random forest algorithm to train our binary and multiple classifiers. We also use 10-fold cross-validation to evaluate the performance of these two options. As shown in Table 6, the performance index of these two options are demonstrated~~respectively~~.

Table 6. Comparison of the two options.

**Malware family**	**MC**			**BCs**		
	**Precision**	**Recall**	**F1-score**	**Precision**	**Recall**	**F1-score**
Dridex	100%	100%	100%	100%	100%	100%
EITest	97.84%	83.81%	90.25%	97.64%	80.00%	87.90%
Emotet	98.28%	94.37%	96.28%	98.49%	94.01%	96.20%
Hancitor	99.56%	99.62%	99.59%	99.49%	99.65%	99.57%
HTBot	100%	81.74%	89.53%	100%	84.35%	91.27%
Nuclear	98.52%	90.00%	94.02%	97.78%	86.00%	91.45%
Razy	100%	100%	100%	100%	100%	100%
Rig	82.60%	64.62%	72.13%	93.06%	58.46%	71.45%
Trickbot	92.23%	94.06%	93.13%	91.31%	94.39%	92.82%
Benign samples	98.62%	99.99%	99.30%	98.63%	100%	99.31%
**Average ~~Accuracy~~ACC**	98.41%			98.36%		
**Time consumption (s)**	108.62			232.51		

~~It can be seen that the~~ The overall performance of the MC is slightly better than that of the BCs, and their average accuracies are 98.41% and 98.36%, respectively. However, due to the mechanism of the second option, which is required to ~~traversing~~ traverse all binary classifiers before obtaining the classification result, the

~~discrimination of consuming time between~~ time consumption difference between the two options is remarkably conspicuous, ~~which~~ as the time consumption of the BCs is twice ~~as much as that of~~ that of the MC. Accounting for the superiorities ~~of~~ in accuracy and efficiency, we adopt the first option (~~multi-classifier~~multiclassifier) to identify the malware family of TLS flows. In a ~~multi-classifier~~multiclassifier, the importance of each feature ~~also can~~ can also be evaluated, and the ~~most important 20~~ 20 most important features are presented in Table 7.

Table 7. The most important 20 features in the ~~Malware Family Classification Model~~malware family classification model.

**Feature description**
Client ~~Cipher Suites~~cipher suites: TLS_ECDHE_RSA_WITH_AES_128_GCM_SHA256
Client ~~Cipher Suites~~cipher suites: TLS_ECDHE_RSA_WITH_AES_256_GCM_SHA384
Client ~~Cipher Suites~~cipher suites: TLS_ECDHE_RSA_WITH_CHACHA20_POLY1305_SHA256
Client ~~Cipher Suites~~cipher suites: TLS_RSA_WITH_RC4_128_MD5
Cipher ~~Suites Number~~suite number: 21 (*new*)
~~Certificates Number~~Certificate number: 1 (*new*)
Server ~~Extensions Number~~extension number: 1 (*new*)
Client ~~Cipher Suites~~cipher suites: TLS_DHE_DSS_WITH_AES_256_CBC_SHA
Client ~~Cipher Suites~~cipher suites: TLS_RSA_WITH_RC4_128_SHA
Client ~~Signature Number~~signature number: 2 (*new*)
Server ~~Name~~ name is not in the top 1 million DNS Alexa results
Server ~~Cipher Suite~~cipher suite: TLS_RSA_WITH_AES_128_CBC_SHA256
Packet ~~Length Distribution~~length distribution: [1490, 1500) (*Statistical features*)
Server ~~Name is~~ name is a random string (*new*)
Packet ~~Length Distribution~~length distribution: [180, 190) (*Statistical features*)
Client ~~Extension Number~~extension number: 5 (*new*)
Client ~~Cipher Suites~~cipher suites: TLS_ECDHE_ECDSA_WITH_CHACHA20_POLY1305_SHA256
Packet ~~Inter-arrival Time Transition Probability Matrix~~interarrival time transition probability matrix: [100, ~~150ms~~150 ms) (*Statis*
Packet ~~Length Transition Probability Matrix~~length transition probability matrix: [980, 990) (*Statistical features*)
Client ~~Extensions Number~~extension number: 3 (*new*)

From Table 7, there are 7 features related to ~~Client Cipher Suites~~client cipher suites, which means that different malware families are ~~intent~~ intended to select different ~~Client Cipher Suites~~such suites. There are still 7 features we newly ~~proposed with~~ propose with a *new* tag in this paper, ~~which demonstrate~~ thus demonstrating again the effectiveness of the features we ~~proposed again.~~ propose. Moreover, TLS handshake features occupy a majority compared to statistical features (~~That is~~that is, 16:4), so we can conclude that the TLS handshake features are more important than statistical features.

Evaluation of the two-layer detection framework

In previous experiments, we ~~have trained the Filtering Model~~ trained the filtering model (a binary classifier) and the ~~Malware Family Classification Model (a multi-classifier~~malware family classification model (a multiclassifier). Combining these two models ~~can constitute~~ constitutes our two-layer detection framework. To verify the efficiencies of the two-layer framework, contrast experiments between it and a single-layer framework ~~is~~ are conducted. As shown in Fig. 8, the ~~Multi-Classifier~~ multiclassifier used in the single-layer framework is the same as the classifier utilized in the second layer of the two-layer framework. The purpose is to ~~prove~~ evaluate whether the two-layer framework ~~could~~ can improve the detection efficiency on the one hand ~~,~~

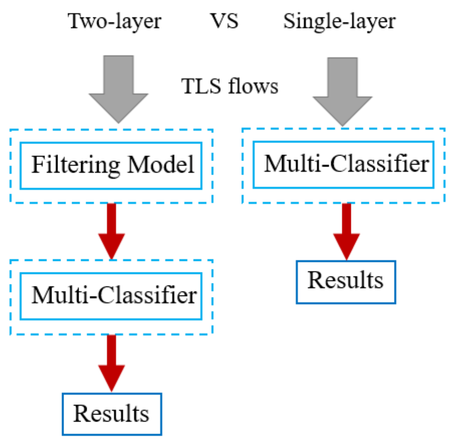

Fig 8. The processing flow of the two frameworks.

and guarantee the detection accuracy on the other hand.

Since benign TLS flows generally account for the majority of flows in a real network environment, it is reasonable to set the number of benign samples to be greater than the number of malicious samples. We prepared a total of 11,000 TLS flows for contrast experiments, including 10,000 new benign samples and 1,000 malicious samples. Before the experiment, we set the threshold of the ~~Filtering Model~~ filtering model to 0.01, as discussed in the ~~Filtering Model. Meanwhile~~filtering model. Moreover, we adopt the random forest algorithm to train both ~~Filtering Model and the Multi-Classifier~~ the filtering model and the multiclassifier in advance. By importing the testing samples into these two detection frameworks, we compare the relative indicators as shown in Table 8.

Table 8. Comparison of the two frameworks.

Malware family	Single-layer			Two-layer		
	Precision	Recall	F1-score	Precision	Recall	F1-score
Dridex	100%	100%	100%	100%	100%	100%
EITest	100%	80.00%	88.89%	100%	80.00%	88.89%
Emotet	93.48%	93.99%	93.73%	93.48%	93.99%	93.73%
Hancitor	98.59%	99.29%	98.94%	98.59%	99.29%	98.94%
HTBot	100%	76.92%	86.96%	100%	76.92%	86.96%
Nuclear	100%	82.00%	90.11%	100%	82.00%	90.11%
Razy	100%	100%	100%	100%	100%	100%
Rig	92.31%	54.55%	68.57%	92.31%	54.55%	68.57%
Trickbot	87.92%	95.62%	91.61%	87.41%	91.24%	89.29%
Benign samples	99.81%	100%	99.91%	99.75%	100%	99.88%
**Average ~~Accuracy~~ACC**	99.51%			99.45%		
**Traversal Times**	11000			11377		
**Time consumption (ms)**	1360.84			722.44		

Table 8 shows that the two-layer framework does not significantly affect the detection results of TLS flows. Although the traversal times of the two-layer framework ~~is~~ are larger than that of the single-layer framework, the time consumption ~~of it decreased~~ decreases by 188% compared to that of the single-layer framework, which

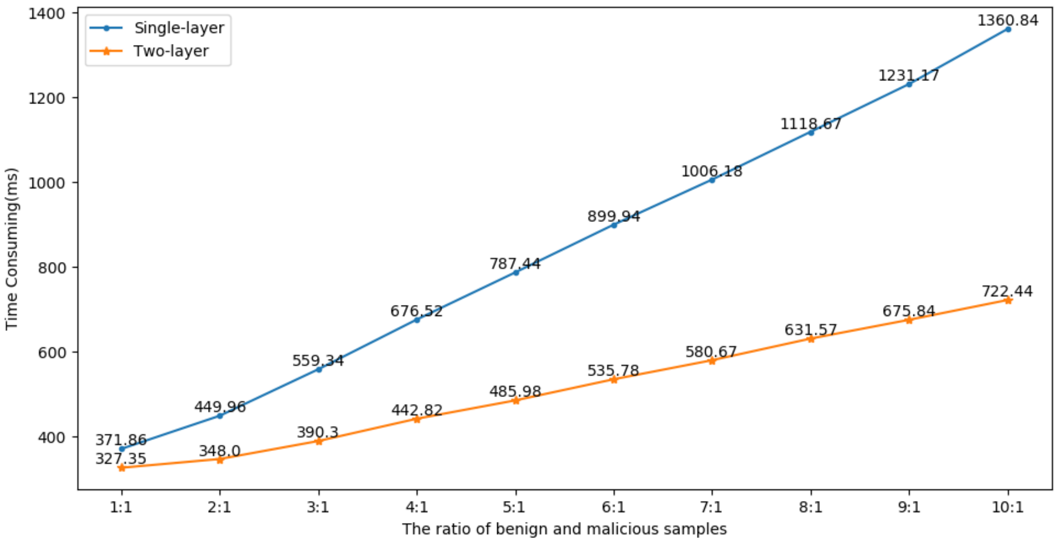

Fig 9. Time consumption at different ~~ratio~~ratios.

means that the efficiency ~~improved~~ improves by 188%. Meanwhile, the average detection accuracy of the two-layer framework reaches 99.45%~~which only produces~~, which produces only a 0.06% loss, which means that the proposed framework also guarantees the detection accuracy. In fact, in the process of the experiment, there are 9623 TLS flows filtered out by the ~~Filtering Model~~filtering model, which accounts for 96.32% of ~~entire~~ all benign samples. At the same time, few malicious TLS ~~flow is~~ flows are filtered out, which proves the reliability of the ~~Filtering Model~~filtering model. Moreover, we can compute the average time consumption of each flow in the ~~Filtering Model and the Multi-Classifier, respectively~~filtering model and the multiclassifier, respectively, as 0.06 ms and 0.12 ms. Substituting these calculated parameters into Ineq. (2), where ~~the~~ r is 96.32%, T_1 is 0.06 ms and T_2 is 0.12 ms, the ~~correctness of the~~ accuracy of Ineq. (2) is substantiated.

We also compare the time consumption at different ratios of benign and malicious samples. At each ratio, we test a total of 10 times and calculate the average time consumption. As shown in Fig. 9, when the ratio is 1:1, the single-layer framework is not much different from the two-layer framework. However, along with the increase in the number of benign samples, the two-layer framework is ~~more and more~~ increasingly advantageous. When the ratio reaches ~~to~~ 10:1, the two-layer framework is nearly twice as fast as the single-layer framework. In the real network environment, since benign TLS flows account for the vast majority ~~,~~ (the ratio is far more than 10:1~~, it is literally grounded to apply~~), application of the two-layer detection framework ~~.~~ is well justified.

~~Summarily~~In summary, we demonstrate that the two-layer detection framework needs to meet certain conditions to improve the detection efficiency of TLS flows. That is~~:~~, 1) the detection efficiency of the coarse classification model in the first layer must be higher than that of the detection models in the second layer; 2) the ratio of flows filtered by the first layer must satisfy Ineq. (2). Otherwise, the improvement ~~of detection efficiency can not~~ in detection efficiency cannot be guaranteed.

We also compare our method with ~~the other~~ 3 ~~methods in~~ other methods in terms of the classification efficiency. The related results are depicted in Fig. 10~~in the revised manuscript,~~, in which the average time consumption of each method at different sample ~~ratio~~ ratios is calculated.

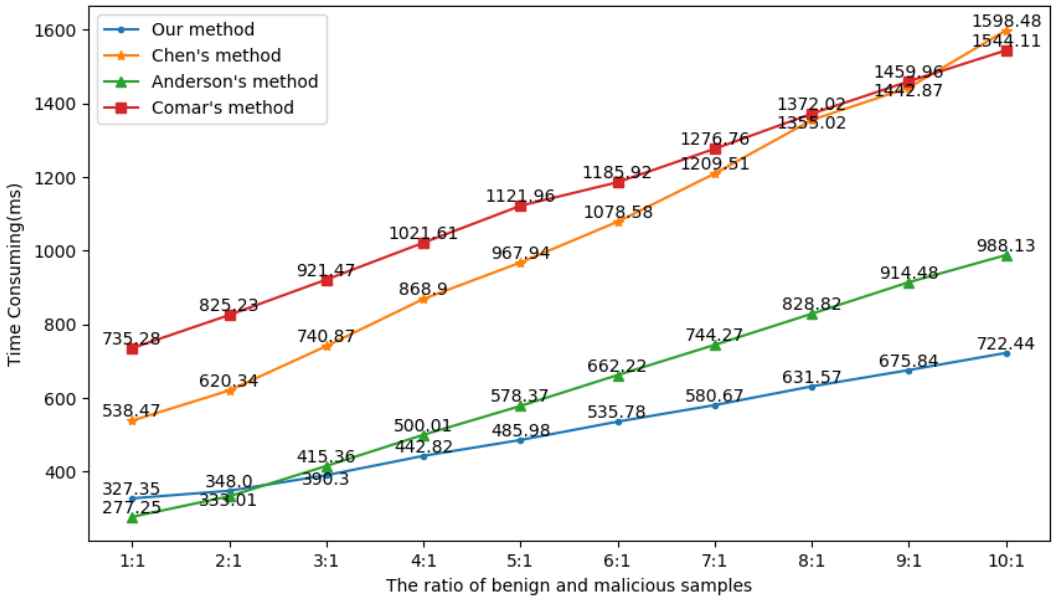

Fig 10. Time consumption among the 4 different methods.

As seen from ~~Fig 10, Anderson~~ the figure, Anderson et al.'s method utilizes a single-layer detection framework, and their method is more efficient than ours when the sample ratio is not more than 2:1. However, ~~it is of low-efficiency~~ their method is of low efficiency when the sample ratio is over 2:1. The reason could be that the number of features they used is less than that in the second layer of our method but more than that in the first layer of our method. Comar's method is based on a two-layer detection framework, in which the first layer is also used to exclude benign flows. ~~But~~ However, the second layer consists of a set of 1-class SVM models to identify a specific malware class, which means that a potential malicious flow needs to traverse all the models before obtaining the classification result. Though the number of features is less than in our method, the time consumption is always higher than ours. Chen's method proposed a triple-layer detection framework~~,~~ ; the additional layer is the second layer, which is used to recognize the attack type. That is, a potential malicious flow needs to be classified twice, which ~~will add~~ adds extra time for detection. Thus, the efficiency of Chen's method is always less than ours.

In summary, though the efficiency of a classifier is ~~largely~~ strongly related to the number of features, our two-layer detection framework is more efficient than other methods that utilize fewer features. There are two reasons~~to achieve this~~: 1) our method ~~utilized a multi-classifier to identify~~ utilizes a multiclassifier to identify the malware family, which is more efficient than ~~a~~ the set of classifiers used by Comar's method. 2) ~~we~~ We used fewer features in the first layer~~,~~ ; as long as the number of features in the ~~Filtering Model~~ filtering model is less than that of other methods (~~like Anderson~~ such as Anderson et al.'s method), our method ~~will be~~ is more efficient than other ~~method with the increase of~~ methods with increasing ratios of benign and malicious samples.

CONCLUSION

The TLS protocol as a kind of cryptographic protocol is increasingly employed to establish the C&C channel by malware. The identification of malicious TLS flows is

becoming an inevitable challenge. In this paper, we proposed a two-layer framework that ~~earned~~ exhibited high accuracy and superior efficiency. The first layer is the ~~Filter Model that~~ filter model, which consists of a ~~binary classification model~~ BC based on a new set of TLS handshake features and is used to filter out benign TLS flows, while the second layer is devised to identify the malware family via both TLS handshake features and statistical features. The reliability of the ~~Filtering Model~~ filtering model is demonstrated via contrast experiments, through which 96.32% of benign TLS flows are filtered out with all ~~the~~ malicious TLS flows ~~being~~ left. Moreover, for dealing with the ~~multi-classification~~ multiclassification problem, we compare the effects between a ~~multi-classifier~~ multiclassifier and a set of binary classifiers under the same feature set. Experiments show that ~~multi-classifier~~ the multiclassifier performs better both in detection efficiency and in detection accuracy. ~~Combining the Filtering Model and the Malware Family Classification Model~~Upon combining the filtering model and the malware family classification model, the high accuracy and superior efficiency of the proposed two-layer detection framework are substantiated by comparison experiments.

During our research, we also ~~observe that the Filtering Model~~ observed that the filtering model has the ability to detect unknown malicious TLS flows. Since we find ~~many discriminations~~ substantial discrimination between benign and malicious TLS flows in the handshake phase, there is a chance to recognize unknown malicious TLS flows. ~~By this token, in the next researchplan, we will redesign the~~ In upcoming research, we plan to redesign further experiments to prove this idea.

References
